# Alpine Snow Algae Microbiome Diversity in the Coast Range of British Columbia

**DOI:** 10.3389/fmicb.2020.01721

**Published:** 2020-07-28

**Authors:** Kurt M. Yakimovich, Casey B. Engstrom, Lynne M. Quarmby

**Affiliations:** Department of Molecular Biology and Biochemistry, Simon Fraser University, Burnaby, BC, Canada

**Keywords:** algae, microbiomes, bacteria, fungi, protists, snow

## Abstract

Snow algae blooms contain bacteria, fungi, and other microscopic organisms. We surveyed 55 alpine snow algae blooms, collecting a total of 68 samples, from 12 mountains in the Coast Range of British Columbia, Canada. We used microscopy and rDNA metabarcoding to document biodiversity and query species and taxonomic associations. Across all samples, we found 173 algal, 2,739 bacterial, 380 fungal, and 540 protist/animalia operational taxonomic units (OTUs). In a previous study, we reported that most algal species were distributed along an elevational gradient. In the current study, we were surprised to find no corresponding distribution in any other taxa. We also tested the hypothesis that certain bacterial and fungal taxa co-occur with specific algal taxa. However, despite previous evidence that particular genera co-occur, we found no significant correlations between taxa across our 68 samples. Notably, seven bacterial, one fungal, and two cercozoan OTUs were widely distributed across our study regions. Taken together, these data suggest that any mutualisms with algae may not be taxon specific. We also report evidence of snow algae predation by rotifers, tardigrades, springtails, chytrid fungi, and ciliates, establishing the framework for a complex food web.

## Introduction

Alpine snow algae microbiomes are threatened by global warming as glaciers and permanent snowfields disappear. Annual snow coverage in the northern hemisphere has decreased by 5–6 days over the last 50 years (Bormann et al., 2018). At the same time, snow algae blooms amplify the rate of snow loss by decreasing snow surface albedo (Thomas and Duval, 1995; Lutz et al., 2016; Ganey et al., 2017), and microbial growth on snow contributes to accelerated snow melt on a global scale (Ganey et al., 2017; Williamson et al., 2020). We seek to understand the microbial diversity that supports these at-risk alpine microbiomes. It is likely that mutualist interactions are essential for algae growth in this extreme and ephemeral habitat, as is the case in aquatic environments (Seymour et al., 2017). Therefore, a key step toward understanding bloom development is to identify the non-algal components of the blooms.

Snow algae blooms can form a patchwork of red, green, or orange snow that covers large areas (Remias et al., 2013b; Lutz et al., 2016; Ganey et al., 2017; Segawa et al., 2018; Davey et al., 2019; Hoham and Remias, 2020). The blooms support rich communities, including viruses, bacteria, fungi, ciliates, and small metazoans (Thomas and Duval, 1995; Duval et al., 1999; Takeuchi et al., 2006; Brown et al., 2015; Hisakawa et al., 2015; Lutz et al., 2016; Segawa et al., 2018; Davey et al., 2019). Snow algae blooms are typically dominated by algae of the phylum *Chlorophyta*, although blooms dominated by phylum *Ochrophyta* have also been reported (Leya, 2013; Remias et al., 2013a, 2020; Hamilton and Havig, 2017; Hoham and Remias, 2020). These bloom-forming algae are widely distributed at low densities in white snow around the globe (Brown and Jumpponen, 2019; Maccario et al., 2019).

Bacteria can promote algal growth through the exchange of metabolites (e.g., vitamins, amino acids, or plant hormones) for fixed carbon (Ramanan et al., 2016; Seymour et al., 2017). These relationships have not been documented in snow algae microbiomes, but there is some evidence for their existence. In a metabolomic study of green snow in Antarctica, Davey et al. (2019) found calystegine, an alkaloid noted for plant-bacterial communication. In a laboratory experiment, Terashima et al. (2017) showed that a *Chloromonas* spp. isolated from snow grew better in the presence of bacteria from a field sample than when plated with an antibiotic. Another study, co-culturing snow bacteria and *Chloromonas brevispina*, showed increased iron containing mineral dissolution, which stimulated algae growth, suggesting bacteria could help snow algae obtain bioavailable iron from mineral dust (Harrold et al., 2018). Although little data exist for algae-fungal mutualisms outside of lichens, metabolite exchange can occur between yeast (*Saccharomyces cerevisiae*) and microalgae (*Chlamydomonas reinhardtii*; Hom and Murray, 2014).

A few studies document snow algae microbiome diversity and geographic variation, but all are limited to a few samples from a limited number of sites (Brown et al., 2016; Lutz et al., 2016; Hamilton and Havig, 2017; Terashima et al., 2017). Lutz et al. (2016) compared the variation in bacterial and algae communities in Arctic environments. They found that sites in Northern Sweden (*n* = 24) had similar bacterial community composition as sites in Svalbard (*n* = 12), but the two locations varied in their relative abundance profiles. In contrast, on Mount Asahi, Japan, patches of red and green snow within a few hundred meters had distinct bacterial profiles (Terashima et al., 2017). Because the algal communities were different (three of the blooms were red and one was green), the authors suggest that different algal species might recruit different bacterial assemblages. Another alpine study, this one in the Austrian Alps by Krug et al. (2020), reports that specific algal genera from red (*n* = 3), green (*n* = 1), and orange (*n* = 1) patches of snow positively correlate with specific bacterial genera.

In the current study, we set out to robustly document the microbial diversity in the mountains of Southwestern British Columbia. Previously, we reported that *Sanguina* and *Chloromonas* dominated distinct blooms and that they were found at different elevations (Engstrom et al., 2020). Based on the work of Terashima et al. (2017) and Krug et al. (2020), we predicted that specific microbial taxa would be associated with the distinct algal blooms.

Over the 2018 melt season, we collected 68 samples from 55 snow algae blooms on 12 different mountains near Vancouver, Canada. Sample sites ranged in elevation from 880 to 2,150 m above sea level, ranging from below and above tree line. We surveyed the algal, protist, metazoan, fungal, and bacterial communities using both microscopy and 16S/18S rDNA metabarcoding. Across all samples, we found 173 algal, 2,739 bacterial, 380 fungal, and 540 protist/animalia operational taxonomic units (OTUs). We did not find any significant co-occurrence patterns, even when examining the specific genera highlighted by Krug et al. (2020). We found only seven bacterial, one fungal, and two glissomonad OTUs that were present in more than 90% of snow algae samples. Also ubiquitous were *Chytridiomycota*, which we observed physically attached to algae cells. We also report photomicrographs of rotifers, tardigrades, and various ciliates with snow algae in their guts.

## Materials and Methods

### Sample Collection

Samples were collected during the 2018 melt season between May and September from 12 mountains near Vancouver, Canada (Supplementary Table 1). Our sampling efforts focused on collecting a wide range of colored snow, including green (*n* = 20), red (*n* = 31), and orange (*n* = 17) from 880 to 2,150 m above sea level. Samples were collected in either a Whirl-Pak® sample bag or a 50 ml centrifuge tube, and the brightest colored snow from a patch was collected until the container was full. In total, 68 samples were analyzed from 55 snow algae bloom sites.

Upon collection, samples were packed together, with extra white snow to ensure they were kept cool until they were returned to the lab. We thawed samples on a lab bench at room temperature, and 1 ml aliquots were fixed in 2% glutaraldehyde for microscopy. Samples were examined at 100x, 630x, and 1,000x magnification on an Axioskop 2 (Zeiss) using differential interference contrast (DIC), and photographed using a Canon EOS T6 camera (Canon, Tokyo, Japan). Samples were then stored at −20°C until processing for DNA extractions.

### DNA Extractions

Samples were thawed at room temperature, and DNA extracted and purified using a chloroform method (Cubero et al., 1999), with a CTAB buffer for the cell lysis steps (3% w/v CTAB, 100 mM Tris pH 8.0, 20 mM EDTA, 2.8 M NaCl, and 1% w/v PVP). We used two different cell lysis methods because after completing the first sequencing run with lysis method 1, we developed lysis method 2, which has fewer steps, thereby reducing points for possible contamination. We also opted for manually grinding cells for cell lysis in method 2 to improve the efficiency of lysing fungi and algae. For 42 samples, we used cell lysis method 1: cells were collected on a 0.2 μm nitrocellulose filter (Sartorius AG) and then the filters were cut into strips and placed in a 2 ml tube filled with 0.1 mm glass beads (Qiagen). Seven hundred microlitres of CTAB extraction buffer was added, and the tubes were sonicated at 40% amplitude for 30 s (Bronson Digital Sonifier 450). After sonication, the samples were shaken for 4.5 min at 5000 rpm in a Precellys 24 tissue homogenizer (Bertin Instruments). Finally, the tubes were incubated at 60°C for 1 h. For 26 samples, we used cell lysis method 2: cells were freeze-dried in the 50 ml collection tubes. The dried pellet was transferred to a 1.5 ml microcentrifuge tube and ground for 1 min by hand using a mini-pestle. Seven hundred microlitres of CTAB extraction buffer was added, and samples incubated for 1 h at 60°C. DNA was quantified using a Qubit (Invitrogen). The lysate from both methods was then carried forward into a chloroform extraction step, and a subsequent EtOH precipitation step after Cubero et al. (1999).

### Amplicon Library Preparation and Sequencing for Metabarcoding

From each sample, we generated amplicons for two targets, the 16S rDNA gene of prokaryotes using the primer set Pro341F (5'-CCT ACG GGN BGC ASC AG-3') and Pro805R (5'-GAC TAC NVG GGT ATC TAA TCC-3'; Takahashi et al., 2014) and the 18S rDNA gene of eukaryotes using the primer pair Euk1181 (5'-TTA ATT TGA CTC AAC RCG GG-3') and Euk1624 (5'-CGG GCG GTG TGT ACA AAG G-3'; Wang et al., 2014). We used a two-step PCR library construction: initial primers of the target gene had a universal adapter attached, generating targeted fragments with the universal 5' and 3' adapter sequence appended to their respective ends. Five microlitres of the original amplicon PCR was placed into a second reaction using primers with the universal adaptor sequence and a unique 6 bp index, resulting in the addition of the 6 bp index sequence to the 3' end of each amplicon (one barcode per sample for all gene targets), and a universal barcode on the 5' end. The cycling conditions were the same for both the 16S and 18S primer pairs. For the first PCR, the reactions were done with a total volume of 25 μl, consisting of 12.5 μl of the Q5 high fidelity 2X MM (New England BioLabs, Inc.), 1.25 μl of each respective forward and reverse primer (10 μM), and 9 μl of ddH_2_O. For the second PCR the ddH_2_O was reduced to 5 μl to compensate for the increased template volume input. For the first PCR, we did an initial denaturation at 98°C for 30 s, followed by 30 cycles of 98°C for 5 s, 58°C for 10 s, and 72°C for 25 s, and then a final extension at 72°C for 2 min. The indexing PCR started with an initial denaturation at 98°C for 30 s, then 10 cycles of 98°C for 10 s, 65°C for 30 s, and 72°C for 30 s with a final denaturation of 72 s for 5 min. After each PCR, the product was purified with the Agencourt AMPure XP kit (Beckman Coulter, Inc.) according to manufacturer specifications. After indexing, they were quantified using a Qubit and standardized for pooling. The pooled library was then loaded and run on a MiSeq (Illumina). Samples used from this study came from two sequencing runs. Amplicons from samples lysed by method 1 were sequenced with a MiSeq V2 kit (Illumina). Amplicons from samples lysed by method 2 were sequenced using a MiSeq V3 kit (Illumina). All raw fastq files were uploaded to the European Nucleotide Archive under the project accession PRJEB34539.

### Sequence Data Processing

Reads for both 16S and 18S rDNA were first demultiplexed using CUTADAPT v2.3 (Martin, 2011), and primer sequences were removed. The reads were quality filtered and merged through the *dada2* default pipeline in R v3.6.1 (Callahan et al., 2016; R Core Team, 2019). For the 16S and 18S rDNA sequences, taxonomy assignments were done by using the Silva small subunit database v132 (Quast et al., 2013), formatted for *dada2*^1^. Taxonomy was assigned using the naïve Bayesian classifier RDP (Wang et al., 2007), implemented by *dada2*. The newly defined genus *Sanguina* (Procházková et al., 2019) is absent from the Silva V132 database; therefore, we aligned all *Chlamydomonas* 18S sequences to the NCBI GenBank database (Clark et al., 2016) using BLAST (Altschul et al., 1990). Sequences were reassigned to *Sanguina* if it was the unambiguous top match (no other matches with identity >92%). Taxonomic assignments were used to define fungal, algal, and other 18S rDNA reads. All analyses were done using relative abundance values unless otherwise stated.

The amplicon sequencing variants (ASVs) output from *dada2* were used to assess the distribution of bacteria and eukaryotes sharing identical 16S or 18S sequences. To further assess the distribution of putative bacterial and fungal species, we grouped 16S and fungal 18S rDNA into OTUs. OTUs were clustered using SWARM v2.0 with the default settings (Mahé et al., 2014), which include a strict sequence clustering threshold of 1 base pair difference during the initial alignment phase. Richness was calculated for each sample by counting the total number of ASVs or OTUs. All subsequent analyses for bacteria and fungi were done on both ASVs and OTUs to assess potential population and species level variation, whereas protist/metazoan communities were assessed with OTUs.

Community data was analyzed in R to ascertain co-occurrences [using Kendall’s tau rank correlations (*𝜏*)] between algae and the fungal, bacterial, and protist communities, using relative abundances. Community trends across elevational gradients were assessed for all microbial groups separately by using a distance-based redundancy analysis (dbRDA) with a Bray-Curtis distance matrix in *vegan* (Oksanen et al., 2017) and with elevation, snow color, and dominant algae genus separately as constraints. The adjusted *R*^2^ values were also calculated to assess the amount of variation explained by each variable. Community structure was also assessed using nonmetric multidimensional scaling (NMDS) and using 95% confidence ellipses to assess community structure by snow color and dominant algae genera. We used the dominant algae genera detected in each sample as a constraint in a dbRDA of the bacterial 16S rDNA data to ask whether dominant algae sequences contributed to structuring the bacterial community. Correlation matrices were computed between all algal-bacterial and algal-fungal ASVs and OTUs to assess co-occurrence patterns. Heatmaps were also constructed in R using the *ggplot2* (Wickham, 2016) and *heatmaply* (Galili et al., 2017) packages, and hierarchical clustering was done in base R to order the samples by similarity along the both axis.

## Results

### Microscopic Documentation of Microbiome Diversity

**Figure 1:**
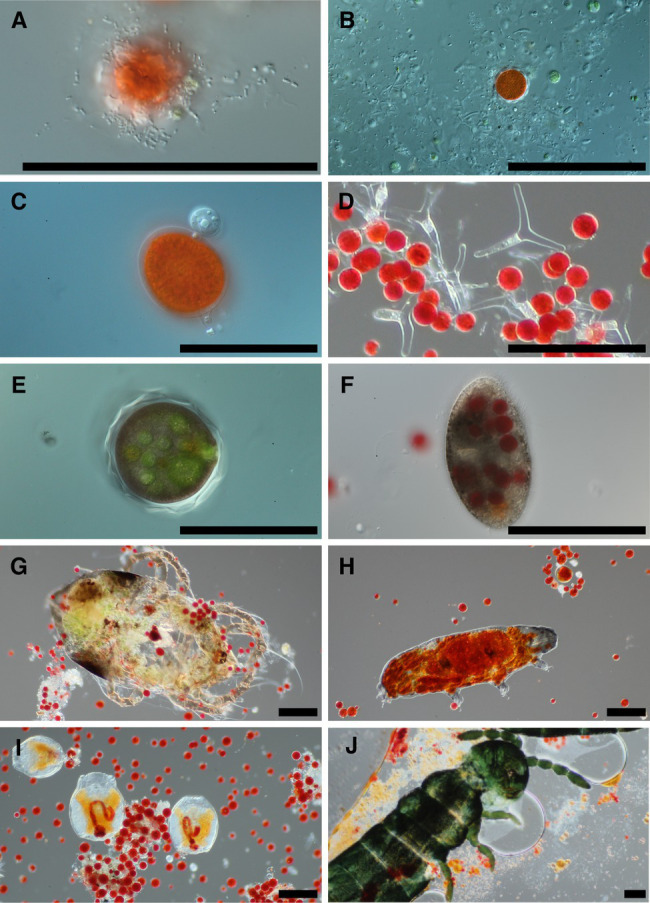
Representative photomicrographs of snow algae bloom samples. Bacteria are seen in the foreground of panel **(A)** with a red pigmented algae cell in the background, and various cells putatively designated as fungi due to size are in panel **(B)**. Panel **(C)** is of two chytrid cells attached to the outside of an algae cell. Panel **(D)** shows a fungal morphological species called *Selenotila nivalis*. Panel **(E)** shows an unidentified micro-eukaryote and panel **(F)** shows a ciliate. Panels **(G–J)** are representative of animals seen in samples, and in order are a mite, tardigrade, rotifers, and a springtail (*Collembola*). All scale bars are 100 μm.

Examination of snow melt samples with light microscopy revealed a diverse set of organisms. Samples often had a visual abundance of prokaryotes in proximity to algae cells (Figure 1A) and in biofilms. Larger fungal cells were often present with various morphologies (Figure 1B). Additionally, small flagellated and larger mature chytrid cells were frequently seen attached to algae cells (Figure 1C). The morphological species called *Selenotila nivalis* were commonly seen (Figure 1D; Light and Belcher, 1968), as was an unidentified micro-eukaryote with a thick outer shell, harboring green spheroids (Figure 1E). We frequently observed presumptive algal predators with their guts full of red-pigmented algae cells. These included diverse ciliates (Figure 1F) and multicellular organisms, tardigrades (Figure 1H), rotifers (Figure 1I), and springtails (Collembola; Figure 1J).

### Metabarcoding Results

**Figure 2:**
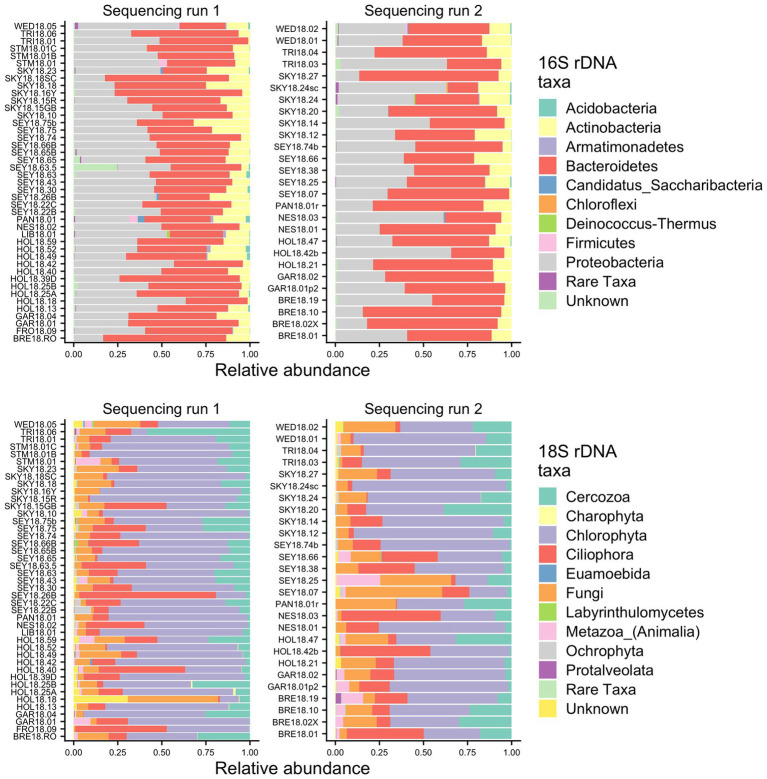
Relative abundance bar plots of all samples from the 16S and 18S rDNA metabarcoding analyses from both sequencing runs. The plots are made based on the taxonomic assignments of the ASVs, and include all ASVs that were above 1% relative abundance in at least one sample. The group “Rare Taxa” includes all ASVs that were below 1% relative abundance and “Unknown” are all ASVs that were unassigned.

To identify the organisms observed by microscopy and to assess the species richness of the microbial groups, we examined the metabarcoding data. Our analysis pipeline outputs a total of 3,742,232 16S rDNA reads with an average of 53,446 (*SD* ± 15,315) reads per sample and 2,392,864 18S rDNA reads with an average of 34,184 (*SD* ± 12,400) reads per sample. Because our data set includes two different sequencing runs, we chose two samples (GAR18.01 and SEY18.74) to compare sequencing run one (MiSeq V2 kit and lysis method 1) with sequencing run two (MiSeq V3 kit and lysis method 2). There were no significant differences in the 16S or 18S rDNA ASV profiles between the batch control samples (Chi-square test *p* = 1; visualized *via* heatmap Supplementary Figure 1). To test for systematic bias, we examined the overall community compositions as a function of sequencing run and found no significant effects (dbRDA: 16S rDNA *R*^2^ = 0.02, 18S rDNA *R*^2^ = 0.04, analyzed *via* NMDS as Supplementary Figure 2). The lack of run bias is further documented in Figure 2, which shows high level taxa for all samples, so we chose to analyze the data from the two sequencing runs together.

**Figure 3:**
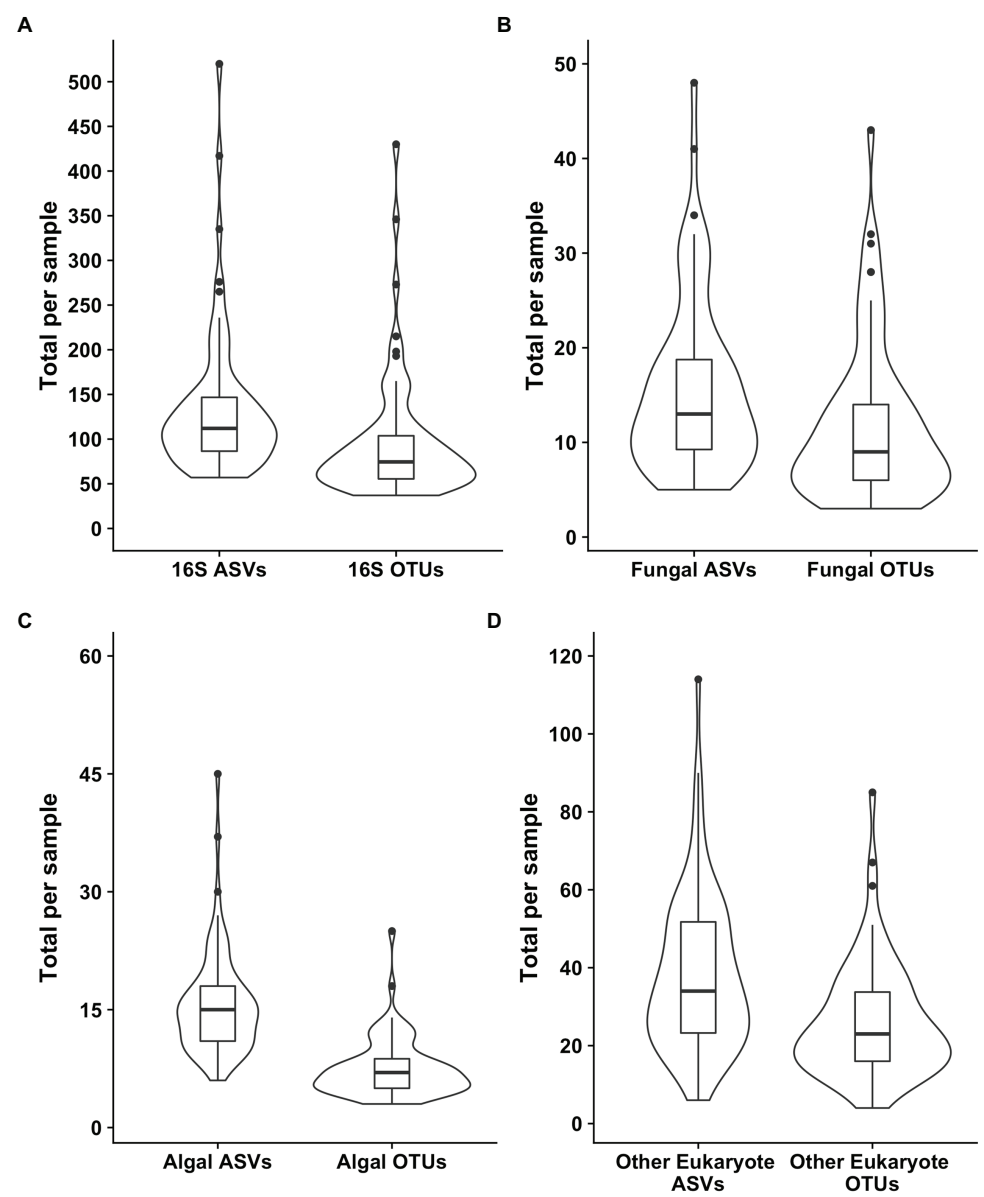
Violin plots of the richness values for both ASVS and those ASVs clustered into operational taxonomic units (OTUs). Plot **(A)** shows the bacterial 16S values, **(B)** the fungal 18S, **(C)** the algal 18S, and **(D)** the “other eukaryotes”, which encompass protists and metazoans.

Samples were generally dominated by three bacterial phyla: *Proteobacteria*, *Bacteroidetes*, and *Actinobacteria* (Figure 2). The 18S sequencing reads from each sample were assigned primarily to *Cercozoa*, *Chlorophyta*, *Ciliophora*, and Fungi (Figure 2). Sequences belonging to methanogens were the only archaea detected and were excluded from analysis due to low read number and infrequent occurrence (<0.04% relative abundance in only four samples). In total, we detected 3,309 bacterial, 380 fungal, 656 protist/animalia, and 173 algal ASVs. When ASVs were grouped into OTUs to estimate species richness, there were 2,739 bacterial, 315 fungal, 540 protist/animalia, and 131 algal OTUs, with on average more ASVs per sample than OTUs (Figure 3).

### Algal Community Composition

**Figure 4:**
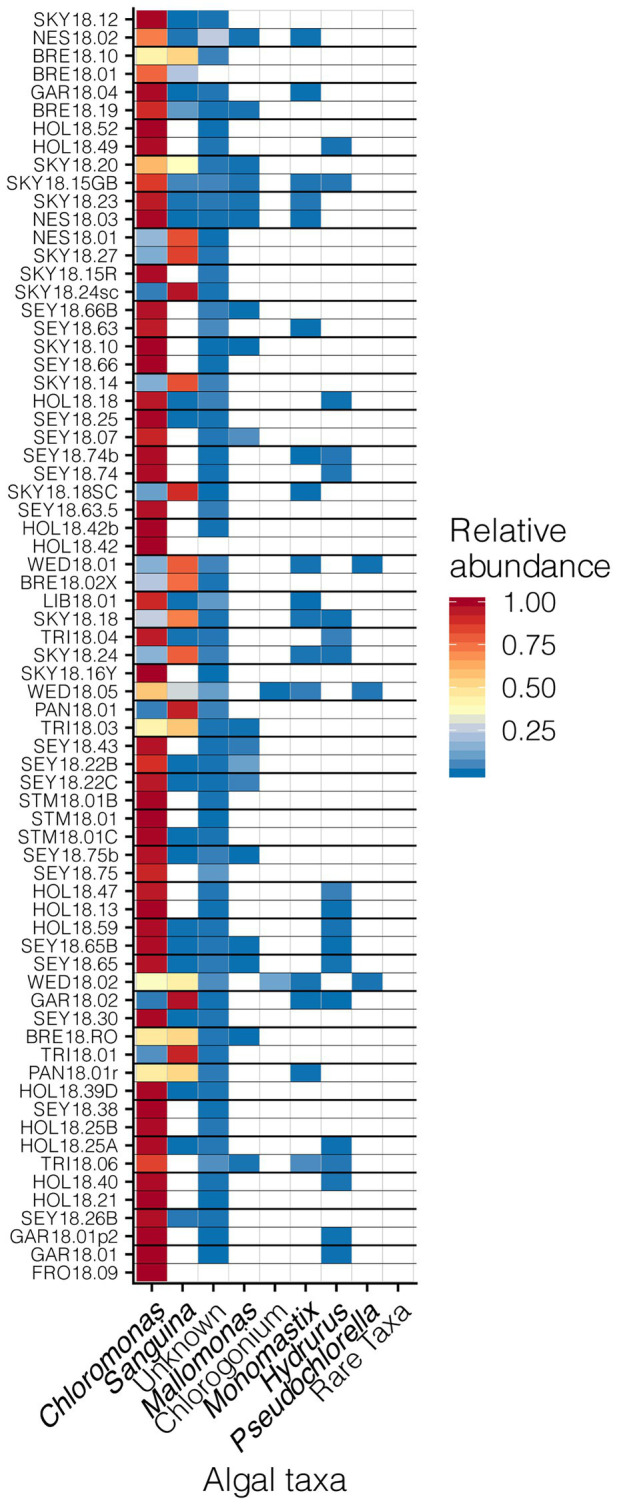
Relative abundances of algal genera across sites presented in a heat map organized by hierarchical clustering analysis. The group “Rare Taxa” includes all ASVs that were below 1% relative abundance and “Unknown” are all ASVs that were unassigned.

Our sampling scheme encompassed a variety of snow conditions across a wide range of elevations on several mountains. We detected 145 ASVs from *Chlorophyta* (*Archaeplastida*), and 28 from *Ochrophyta* (Stramenopiles). In addition to greater sequence diversity, the *Chlorophyta* were more abundant with 114x more total sequencing reads than *Ochrophyta*. The *Chlorophyta* were comprised mostly of *Chlorophyceae*, with some *Trebouxiophyceae* and *Mamiellophyceae*. All snow samples were dominated by either *Chloromonas* spp. or *Sanguina* spp.; *Chloromonas*-dominated sites were the most common (Figure 4). The families of *Ochorophyta* detected included *Chrysophyceae*, *Chrysocapsales*, *Mallomonadaceae*, and *Xanthophyceae*. For a higher resolution analysis of the algal communities within our study region see Engstrom et al. (2020).

### Bacterial Communities

**Figure 5:**
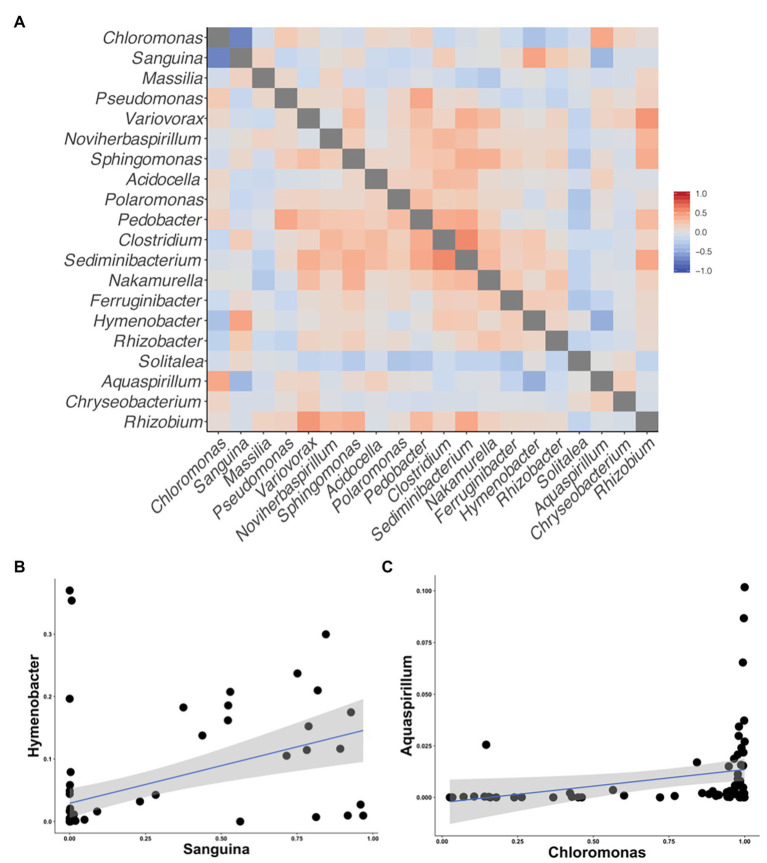
Correlation matrices of the taxa reported by Krug et al. (2020) to be correlated with snow algal taxa in the Alps. All correlations were calculated using Kendall’s tau rank correlations (*𝜏*). **(A)** A heatmap of the correlations between taxa on the axis. **(B)** A scatterplot between *Sanguina* and the highest correlated bacterial genus (*𝜏* = 0.45) from the heatmap in **(A)**. **(C)** A scatterplot between *Chloromonas* and highest correlated bacterial genus (*𝜏* = 0.43) from the heatmap in **(A)**.

Although we found similar phlyum-level bacterial diversity at all sites (see Figure 2), we wanted to examine the community structure at ASV and OTU level to test for specific co-occurrence, particularly of bacteria and fungi with algae. We used both unconstrained (NMDS) and constrained (dbRDA) ordinations to test variation in the community structure. Both NMDS and dbRDA found that the bacterial community structure did not correspond to that of the algae community (dbRDA constrained by dominant algal genus; ASVs *R*^2^ = 0.07: OTUs *R*^2^ = 0.07; NMDS Supplementary Figure 3). We constructed correlation matrices of bacterial 16S rDNA ASVs/OTUs against algae 18S rDNA ASVs/OTUs and did not find any significant correlates (all Kendall’s tau rank correlations; *𝜏* < 0.5). We then looked for correlations between bacterial genera, and the two dominant algal genera, *Chloromonas* and *Sanguina*, and again found no correlations (<0.5 for all). Next, we examined the bacterial-algal genera reported by Krug et al. (2020) to be positively correlated in snow algal blooms found in the Austrian Alps and found no significant relationships (Figure 5A). The strongest correlations for *Sanguina* and *Chloromonas* were with *Hymenobacter* (*𝜏* = 0.45) and *Aquaspirillum* (*𝜏* = 0.43), respectively, but visualized as scatterplots, these correlations were largely driven by a handful of sites and were not representative of all samples (Figures 5B,C). The bacterial community showed little variation between samples with different snow colors, as revealed by a dbRDA constrained by snow color (ASVs *R*^2^ = 0.05; NMDS Supplementary Figure 4), and bacterial OTUs showed similarly low variation (OTUs *R*^2^ = 0.07; NMDS Supplementary Figure 4). Therefore, according to the dbRDAs, large amounts of the variation in the community composition of ASVs or OTUs could be explained neither by dominant algae taxa nor by snow color. Nor did we find any large shifts in bacterial communities based on changes in elevation using either an NMDS analysis or a dbRDA analysis with elevation as a constraining variable (*R*^2^ = 0.11 for ASVs, and *R*^2^ = 0.11 for OTUs, Supplementary Figure 5). There were no regional patterns based on mountain when analyzed by NMDS (Supplementary Figure 6).

**Figure 6:**
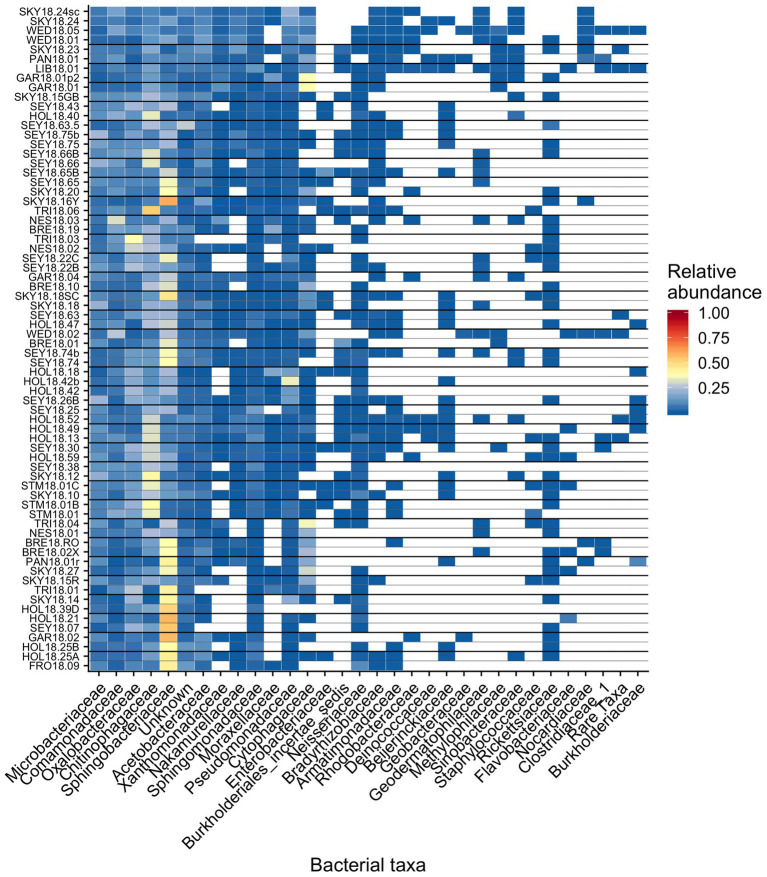
Relative abundance of the most prevalent bacterial families across sites, presented as a heat map organized by hierarchical clustering analysis. The group “Rare Taxa” includes all ASVs that were below 1% relative abundance and “Unknown” are all ASVs that were unassigned.

**Figure 7:**
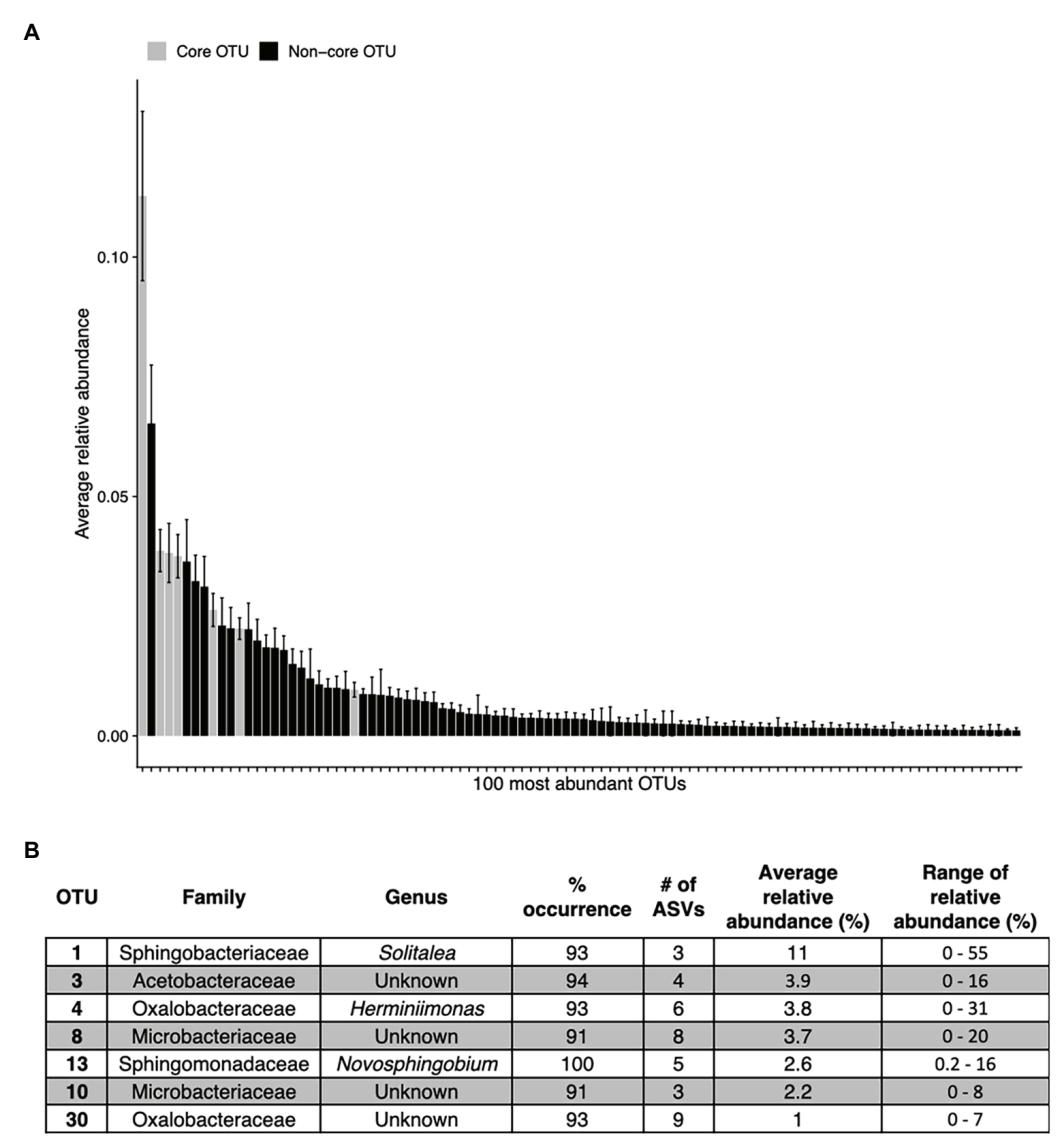
The top abundant bacterial OTUs in our study including seven that are widespread. Panel **(A)** shows the average relative abundance across all 68 samples for the top 100 most abundant OTUs, error bars are standard error. Panel **(B)** shows the taxonomic assignments for each widespread OTU (in >90% of samples). These are shown as gray bars in panel **(A)**. Percentage of sites indicates the occurrence of each OTU; # of ASVs refers to the total number of ASVs constituting each OTU. The OTUs in **(B)** are ordered as they appear left to right in **(A)**.

Across all samples, 10 bacterial families (out of 120 total families) were prevalent, occurring in at least 75% of samples with six being found in all samples (Figure 6 and Table 1). While most OTUs were only found in a few samples, seven OTUs were widespread, occurring in at least 61 out of our 68 samples (≥90%; Figure 7; Supplementary Figure 7). The seven widespread OTUs did not comprise the top seven most abundant OTUs on average (Figure 7A) and, therefore, were not necessarily representative of the dominant community in any one sample. Several predominant families were not represented by any of the widespread OTUs. These include *Chitonphagacea*, *Comamonadaceae*, *Cytophagaceae*, *Neisseriaceae*, and *Pseudomonadaceae* (Figures 6, 7 and Table 1).

**Table 1:** Bacterial families found in >75% of samples.

Family	% occurrence
Acetobacteraceae	96
Chitinophagaceae	100
Comamonadaceae	100
Cytophagaceae	79
Microbacteriaceae	100
Neisseriaceae	79
Oxalobacteraceae	100
Pseudomonadaceae	99
Sphingobacteriaceae	100
Sphingomonadaceae	100

### Fungi and Protists

**Figure 8:**
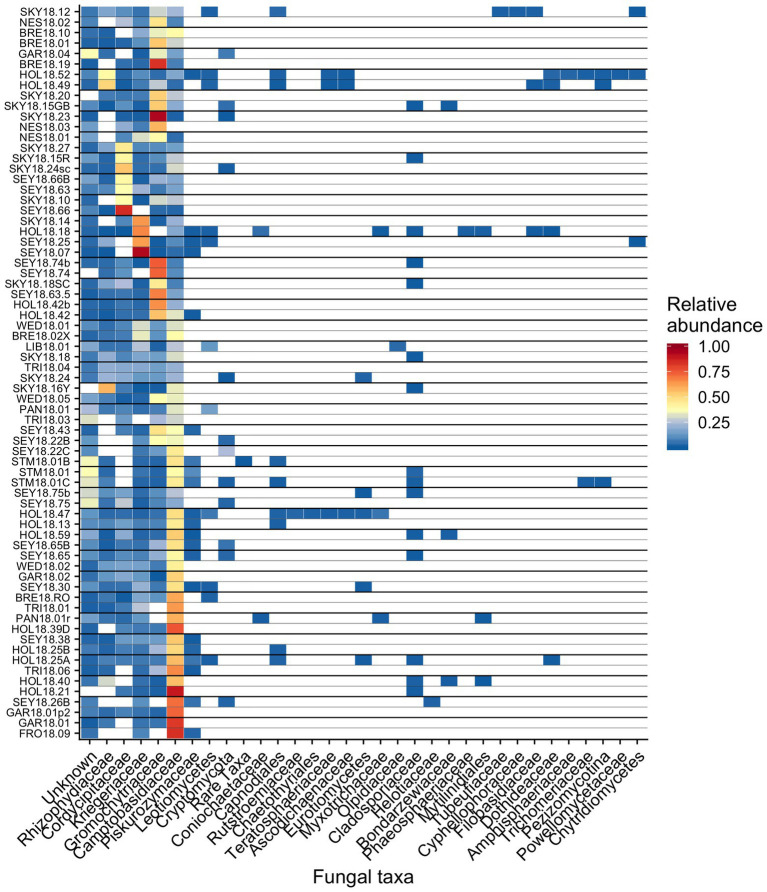
Relative abundance of the most prevalent bacterial families across sites shown as a heatmap organized by hierarchical clustering analysis. The group “Rare Taxa” includes all ASVs that were below 1% relative abundance and “Unknown” are all ASVs that were unassigned.

Out of 74 detected fungal families, the most common in our samples included *Camptobasidiaceae*, *Cordycipitaceae*, *Gromochytriaceae*, *Kriegeriaceae*, and *Rhizophydiaceae* (Figure 8). Fungal OTU 1 (assigned to *Camptobasidium*) was found in all samples but one (NES18.03). Fungal OTU 1 comprises an average of 29% (*SE* ± 1.9%) of detected fungal sequences. We commonly saw *Chytridiomycota*, which were morphologically identified as translucent spheroids attached to algal cells (Figure 1C). In total, there were 22 *Chytridiomycota* OTUs belonging to the groups *Gromochytriaceae* (found in 87% of samples) and *Rhizophydiales* (found in 69% of samples). Although no single *Chytridiomycota* OTU was widespread, as a group they were detected in most samples (Table 2).

**Table 2:** Eukaryotic taxa found in snow algae blooms *via* 18S rDNA sequencing.

OTU#	Phyla or Family	Genus	% of sites	# of ASVs
1	Camptobasidiaceae	*Camptobasidium*	97	5
-	Chytridiomycota	-	94	78
3	Glissomonadida	*Heteromita*	97	15
7	Glissomonadida	Unknown	91	7
-	Rotifera	-	63	23
-	Acari	-	25	21
-	Collembola	-	26	16

Shown are the widespread fungi, protists, and multicellular eukaryotes (Animalia). Taxonomic assignments were based on the most abundant ASVs within an operational taxonomic unit (OTU).

To examine fungal co-occurrence with algae, we created a correlation matrix of the relative abundances of the fungal families against *Chloromonas* and *Sanguina*. We found no significant correlations (*𝜏* < 0.5). The fungal community composition showed little variation that corresponded to changes in elevation (OTU *R*^2^ = 0.04, ASV *R*^2^ = 0.04; NMDS Supplementary Figure 8), dominant algae genus (OTU *R*^2^ = 0.03, ASV *R*^2^ = 0.03; NMDS Supplementary Figure 9), or snow color (OTU *R*^2^ = 0.03, ASV *R*^2^ = 0.03; NMDS Supplementary Figure 10).

**Figure 9:**
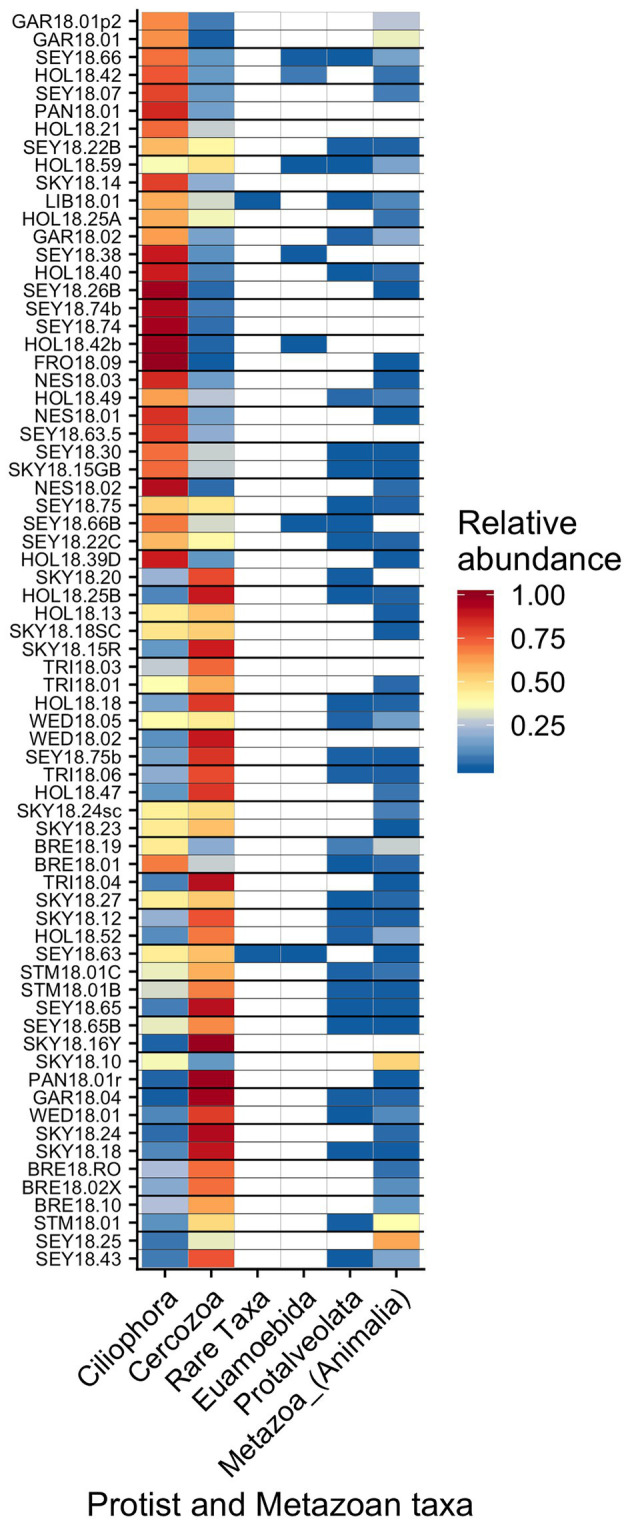
Relative abundance of the remaining eukaryotic groups across sites presented a heatmap organized by hierarchical clustering analysis. The group “Rare Taxa” includes all ASVs that were below 1% relative abundance and “Unknown” are all ASVs that were unassigned.

Other taxa detected by 18S sequencing included cercozoans, ciliates, and metazoans (Figure 9). Two cercozoan OTUs were widespread (Table 2). The remaining ASVs were primarily attributed to *Cercozoa* (290 additional OTUs) and *Ciliophora* (115 OTUs), with each group represented in every sample. Common taxa of Animalia included Rotifers (of the classes *Bdelloidea* and *Monogononta*), *Collembola* (springtails), and *Acari* spp. (mites), which taken together were detected in 72% of our samples (Table 2). Interestingly, *Cercozoa* and *Ciliophora* tended to be dominant at different sites (Figure 9). When we examined the genera driving this pattern, we found a negative correlation between the cercozoan *Heteromita* and the ciliate *Stokesia* (*𝜏* = −0.48). The protist/metazoan community showed little variation across elevation (dbRDA for OTU *R*^2^ = 0.02, OTU *R*^2^ = 0.03; NMDS Supplementary Figure 11), dominant algal genus (dbRDA for OTU *R*^2^ = 0.03, OTU *R*^2^ = 0.03; NMDS Supplementary Figure 12), or sample snow color (dbRDA for OTU *R*^2^ = 0.01, OTU *R*^2^ = 0.02; NMDS Supplementary Figure 13).

## Discussion

We found an abundance of bacteria, fungi, protists, and metazoans thriving alongside algae in the mountains of southwestern British Columbia. Key bacterial families and a few specific OTUs were predominant across all samples. This was a robust pattern observed across a total of 68 samples from 55 unique blooms that encompassed red, green, and orange snow. We failed to observe the co-occurrence of any specific bacteria-alga combinations. Our sequencing data provide insight into the taxa involved at each node of a hypothetical snow algae food web (Supplementary Figure 14) and create a framework for future work exploring trophic connections.

Bacteria are often noted as vital components near the base of food webs (Fenchel, 2008), turning nutrients and various carbon sources into biomass and making them bioavailable for higher trophic levels. Even though algae are likely the dominant source of fixed carbon in their microbiome, there may be other sources of bioavailable carbon that may enter the food web *via* bacteria (and possibly fungal decomposers). Black carbon particulate deposited from the atmosphere onto snow surfaces can spur microbial growth (Malits et al., 2015; Weinbauer et al., 2017). In addition, decomposition of detritus has been noted as an important carbon source in aquatic food webs (Matveev and Robson, 2014) and likely plays a role in the cycling of carbon within the snow algae microbiome. At present, the relative contribution of each carbon source to the snow algae microbiome is unknown.

Seymour et al. (2017) argue that algal-bacterial mutualistic relationships may be more prevalent than antagonistic ones, as these organisms work together to survive in a wide range of environments. The bacteria that were widespread in our study, all of which had populations larger than the vast majority of detected bacteria, may include mutualist partners of snow algae. Previous work reported many of the same bacterial taxa (Figures 6, 7B and Table 1) and widely distributed OTUs (Figure 7B) from both polar and alpine snow algae blooms, including *Sphingobacteriaceae*, *Chitonphagaceae*, and *Cytophagia* (Brown et al., 2015; Lutz et al., 2016; Hamilton and Havig, 2017; Terashima et al., 2017; Davey et al., 2019). Various *Proteobacteria* are also often reported, including *Oxalobacteraceae* (e.g., *Glaciimonas*), *Comamonadaceae* (e.g., *Polaromonas*), and *Pseudomonas* (Lutz et al., 2016; Hamilton and Havig, 2017; Terashima et al., 2017; Davey et al., 2019). The most abundant OTU in our study was assigned to the genus *Solitalea* (Figure 7). Previously, a comparative analysis of 18 *Sphingobacteriaceae* strains revealed diversity in genes related to cold adaptations, osmotic regulation, and secondary metabolisms (N and C species processing; Shen et al., 2017). As *Sphingobacteriaceae* are a prolific group in terms of abundance and distribution, they likely play an important role in biogeochemical cycling within the snow algae microbiome. Genomic analysis of these snow-borne bacteria will be necessary to differentiate their metabolic niches within the microbiome.

The only bacterial OTU found in all samples belonged to the genus *Novosphingobium*, which contains species capable of degrading various aromatic compounds (Wang et al., 2018). Interestingly, while the family *Sphingomonadaceae* (although not *Novosphingobium* specifically) has been reported in the Alps by Krug et al. (2020), it was not reported by Terashima et al. (2017) on Mount Asahi in Japan or by Hamilton and Havig (2017) on Mountains in the Pacific Northwest of the USA. Neither *Novosphingobium* nor *Sphingomonadaceae* were reported in studies from the Arctic (Lutz et al., 2016) or Antarctic (Davey et al., 2019) regions. But using transects across Fennoscandia and Colorado, USA, Brown and Jumpponen (2019) found that *Novosphingobium* was widely distributed in white snow on both continents. While regional variation could account for the absence of *Novosphingobium* at some sites, we propose it may be widely distributed, but because it is low abundance (Figure 7), it may go unreported in studies focused on between-site differences.

In a study at two sites in the Austrian Alps, Krug et al. (2020) reported correlations between *Chloromonas* and the bacterial genera *Ferruginibacter* and *Hymenobacter* and *Chlamydomonas* (*Sanguina* in our study) with *Aquaspirillum*, *Chryseobacterium*, and *Rhizobium*. We did not find these associations in our 68 samples snow algae samples, from 55 sites (Figure 5A). These results suggest that the co-occurrence patterns reported by Krug et al. (2020) are unlikely to be indicative of taxa or species-specific mutualisms. The general lack of specific algal-bacteria associations in our study suggests a paucity of obligate mutualisms. However, based on the importance of bacterial-algal mutualisms in other aquatic systems (Seymour et al., 2017), it is possible that non-specific mutualisms are at work in snow algae microbiomes.

It has been hypothesized that fungi play an important role in algae blooms because they are enriched relative to adjacent white snow (Brown et al., 2015). Many snow-borne fungi found in blooms are presumptive yeasts (Brown et al., 2015; Davey et al., 2019). A widespread OTU in our study belongs to the genus *Camptobasidium*, which is a member of *Kriegeriales*, an order with known yeast forms. The type of symbiotic relationships, if any, these fungi have within the microbiome is unknown, but they likely metabolize carbon fixed by algae. Similar fungal taxa can also be found in other cryosphere environments, such as on ice sheets in Greenland, where Perini et al. (2019) found the family *Microbotryomycetes* in super glacial water, sediment, and water from cryoconite holes. *Microbotryomycetes* was widespread and abundant in our study, primarily represented by *Camptobasidiaceae* and *Kriegeriaceae* (Figure 8).

We observed chytrid fungi attached to snow algae (Figure 1), reminiscent of the chytrid *Gromochytrium mamkaevae*, a known parasite of freshwater algae (Figure 1; Karpov et al., 2014). Gromochytriaceae was prevalent in our 18S metabarcoding data (Figure 8) and is known to have over 225 aquatic species that are primarily algae parasites within the genus *Rhizophydium* alone (Karpov et al., 2014). Our observations are consistent with those made in alpine snows in Colorado, where chytrids have been noted to attach to algal cells (Stein and Amundsen, 1967). Therefore, it is likely that *Chytridiomycota* play a role in the carbon cycling within a bloom by parasitizing algae and are part of the snow food web (Naff et al., 2013).

Many of the fungal and bacterial taxa found in our snow algae samples have also been detected in white snow (Brown and Jumpponen, 2019), as well as in other alpine and polar snow algae blooms (Lutz et al., 2016; Hamilton and Havig, 2017; Terashima et al., 2017). Particularly, OTUs from the families *Sphingobacteriaceae*, *Pseudomonadaceae*, *Chitinophagaceae*, and *Oxalobacteraceae* were dominant in white snow samples from Colorado and Fennoscandia, and found in polar snow packs within snow algae blooms (Lutz et al., 2016; Brown and Jumpponen, 2019; Davey et al., 2019). The overlap in bacterial taxa between these diverse and globally distributed studies indicates the global distribution of these taxa in both white snow and red snow. Brown and Jumpponen (2019) found several fungal genera to be widespread (i.e., *Cryptococcus*, *Kabatiella*, and *Sydowia*) that were not in our study, indicating possible larger scale geographical variation not captured by our study.

Predation plays an important role in the structure of food webs (Fox, 2007), but predator-prey relationships have not yet been explored in snow algae microbiomes. Taxa of protists that could act as algae predators have been noted in blooms before, such as euglenids, nematodes, and ciliates (Bidigare et al., 1993; Ling and Seppelt, 1993; Duval et al., 1999; Davey et al., 2019), but until now, there has been no photographic documentation of protists preying on snow algae cells (Figure 1).

As anticipated based on our microscopy, sequencing revealed an abundance of protist sequences present in all samples. Several OTUs were assigned as *Heteromita* spp. with one found in all but one sample: *Heteromita globosa* is closely related to our widely distributed protist OTU 3, which is a bacterivorous testate amoeba that has been isolated from both temperate and cold environments. A second protist OTU that was widely distributed in our samples was a glissomonad, of undetermined genus. Because our collection protocol was not optimized for the collection of mobile metazoans, which are larger than algae and therefore occur at lower densities, our data set may underestimate their true distributions.

## Conclusion

Over the course of a single season and across 12 mountains near Vancouver, B.C. We documented the diversity of microbial communities within snow algae microbiomes. We found that variation in the bacterial and fungal communities did not reflect variation in the algal communities, nor did they change with elevation. We found similar bacterial taxa (phyla and families) as previously found in other alpine and in polar snow algae blooms. Importantly, we found that certain families and some OTUs were prevalent across an entire region. Unicellular predators, predominantly a testate amoeba and another unidentified cercozoan were found in all samples. Other widespread taxa included *Chytidiomycota*, *Rotifera*, *Acari* (mites), and *Collembola* (springtails). We found no evidence to support specific associations between algae and bacteria.

## Data Availability Statement

The raw sequence data presented in this study are publicly accessible in the European Nucleotide Archive under accession number PRJEB34539.

## Author Contributions

KY, CE, and LQ all contributed to the design of this project and collection of field samples. KY and CE did the lab work, and KY analyzed the data and wrote the manuscript with input from all authors. All authors contributed to the article and approved the submitted version.

### Conflict of Interest

The authors declare that the research was conducted in the absence of any commercial or financial relationships that could be construed as a potential conflict of interest.

## Footnotes

^1^https://benjjneb.github.io/dada2/training.html
