# Supplementary material for: Alpine Snow Algae Microbiome Diversity in the Coast Range of British Columbia
Source: Front Microbiol. 2020 Jul 28;11:1721. doi: 10.3389/fmicb.2020.01721 (PMC7485462; doi:10.3389/fmicb.2020.01721)
Supplement: Supplementary file 1 [file Data_Sheet_1.PDF]

Supplemental data for Yakimovich et al. 2020.

Table 1: Collection data on the samples that were sequenced. Samples taken at different depth profiles are indicated by sequential lettering in the "sample id" column (e.g. HOL18.25A was taken from the surface, and HOL18.25B was taken from just below HOL18.25A).

sample id	date	long	lat	elevation	mountain	sequencing run	lysis method
BRE18.01	2018-07-21	50.039471	-123.18987	1673.3	Mount Brew	2	2
BRE18.02X	2018-07-21	50.039336	-123.19068	1678.5	Mount Brew	2	2
BRE18.10	2018-07-21	50.039766	-123.19066	1678.3	Mount Brew	2	2
BRE18.19	2018-07-22	50.039402	-123.19094	1681.5	Mount Brew	2	2
BRE18.RO	2018-07-22	50.039402	-123.19094	1680.9	Mount Brew	1	1
FRO18.09	2018-05-16	49.38137	-123.05699	1171	Mount Fromme	1	1
GAR18.01	2018-07-27	NA	NA	1600	Mount Garabaldi	1	1
GAR18.01	2018-07-27	NA	NA	1600	Mount Garabaldi	2	2
GAR18.02	2018-07-27	49.826167	-122.96373	1553.6	Mount Garabaldi	2	2
GAR18.04	2018-07-27	NA	NA	1550	Mount Garabaldi	1	1
HOL18.13	2018-05-14	NA	NA	900	Hollyburn Mountain	1	1
HOL18.18	2018-05-14	49.387596	-123.18828	891.5	Hollyburn Mountain	1	1
HOL18.21	2018-05-18	49.398471	-123.18404	1246.3	Hollyburn Mountain	2	2
HOL18.25A	2018-05-18	49.396796	-123.18253	1219.2	Hollyburn Mountain	1	1
HOL18.25B	2018-05-18	49.396818	-123.18257	1219.8	Hollyburn Mountain	1	1
HOL18.39D	2018-05-23	49.398442	-123.18406	1246.4	Hollyburn Mountain	1	1
HOL18.40	2018-06-11	49.387843	-123.18826	886.3	Hollyburn Mountain	1	1

HOL18.42	2018-06-11	49.383601	-123.18439	998.3	Hollyburn Mountain	1	1
HOL18.42b	2018-06-11	49.383575	-123.18439	998.3	Hollyburn Mountain	2	2
HOL18.47	2018-06-14	49.380726	-123.18737	958.7	Hollyburn Mountain	2	2
HOL18.49	2018-06-14	49.384296	-123.18251	1021.7	Hollyburn Mountain	1	1
HOL18.52	2018-06-19	49.383597	-123.18434	998.1	Hollyburn Mountain	1	1
HOL18.59	2018-07-03	49.386621	-123.18101	1058	Hollyburn Mountain	1	1
LIB18.01	2018-08-06	48.515831	-120.65631	1800	Liberty Bell Mountain	1	1
NES18.01	2018-07-14	49.033348	-121.53303	1957.8	Nesakawatch Spires	2	2
NES18.02	2018-07-14	49.033518	-121.53312	1956.5	Nesakawatch Spires	1	1
NES18.03	2018-07-14	49.034049	-121.53376	1926.1	Nesakawatch Spires	2	2
PAN18.01	2018-09-08	49.956464	-123.00978	2048.70239	Panorama Ridge	1	1
PAN18.01r	2018-09-08	49.956464	-123.00978	2048.70239	Panorama Ridge	1	1
SEY18.07	2018-05-22	49.387752	-122.94252	1364.4	Mount Seymour	2	2
SEY18.22B	2018-06-05	49.387693	-122.94237	1366.9	Mount Seymour	1	1
SEY18.22C	2018-06-05	49.387693	-122.94237	1366.9	Mount Seymour	1	1
SEY18.25	2018-06-05	49.383608	-122.94175	1257.2	Mount Seymour	2	2
SEY18.26B	2018-06-05	49.380193	-122.9421	1221.8	Mount Seymour	1	1
SEY18.30	2018-06-05	49.375244	-122.94642	1142.4	Mount Seymour	1	1
SEY18.38	2018-06-14	49.37437	-122.95023	1089.4	Mount Seymour	2	2
SEY18.43	2018-06-14	49.387745	-122.94233	1366.9	Mount Seymour	1	1
SEY18.63	2018-06-28	49.375315	-122.94636	1143.8	Mount Seymour	1	1

SEY18.63.5	2018-07-04	49.380063	-122.942	1222.6	Mount Seymour	1	1
SEY18.65	2018-07-04	49.384893	-122.93831	1220.4	Mount Seymour	1	1
SEY18.65B	2018-07-04	49.384893	-122.93831	1220.4	Mount Seymour	1	1
SEY18.66	2018-07-04	49.387693	-122.94131	1382.1	Mount Seymour	2	2
SEY18.66B	2018-07-04	49.387693	-122.94131	1382.1	Mount Seymour	1	1
SEY18.74	2018-07-19	49.386561	-122.94191	1372	Mount Seymour	1	1
SEY18.74	2018-07-19	49.386561	-122.94191	1372	Mount Seymour	2	2
SEY18.75	2018-07-19	49.387659	-122.94141	1380.8	Mount Seymour	1	1
SEY18.75b	2018-07-19	49.387659	-122.94141	1380.8	Mount Seymour	1	1
SKY18.10	2018-05-20	49.65132	-123.08532	1135.9	Sky Pilot Mountain	1	1
SKY18.12	2018-05-20	49.655358	-123.10622	945.5	Sky Pilot Mountain	2	2
SKY18.14	2018-06-20	49.635188	-123.09231	1804.8	Sky Pilot Mountain	2	2
SKY18.15GB	2018-07-07	49.645055	-123.08717	1388.7	Sky Pilot Mountain	1	1
SKY18.15R	2018-07-07	49.645055	-123.08717	1388.7	Sky Pilot Mountain	1	1
SKY18.16Y	2018-07-07	49.644212	-123.08823	1400	Sky Pilot Mountain	1	1
SKY18.18	2018-07-07	49.639713	-123.09057	1654.3	Sky Pilot Mountain	1	1
SKY18.18SC	2018-07-07	49.639713	-123.09057	1654.3	Sky Pilot Mountain	1	1
SKY18.20	2018-07-07	49.63924	-123.09071	1666.7	Sky Pilot Mountain	2	2
SKY18.23	2018-07-07	49.641645	-123.0924	1574.3	Sky Pilot Mountain	1	1
SKY18.24	2018-08-26	49.636013	-123.0936	1771.65088	Sky Pilot Mountain	1	1
SKY18.24	2018-08-26	49.636013	-123.0936	1771.65088	Sky Pilot Mountain	2	2

SKY18.27	2018-08-26	49.636308	-123.09119	1742.80481	Sky Pilot Mountain	2	2
STM18.01	2018-06-09	49.428094	-123.20676	1354	St. Marks Summit	1	1
STM18.01B	2018-06-09	49.428094	-123.20676	1354	St. Marks Summit	1	1
STM18.01C	2018-06-09	49.428094	-123.20676	1354	St. Marks Summit	1	1
TRI18.01	2018-06-23	50.009583	-123.25212	1699.3	Tricouni Peak	1	1
TRI18.03	2018-06-23	50.005466	-123.25743	1549.9	Tricouni Peak	2	2
TRI18.04	2018-06-23	50.004854	-123.25849	1550	Tricouni Peak	2	2
TRI18.06	2018-06-23	49.993741	-123.26299	1267.1	Tricouni Peak	1	1
WED18.01	2018-08-13	50.150074	-122.79696	2136	Wedge Mountain	2	2
WED18.02	2018-08-13	50.150285	-122.79655	2125	Wedge Mountain	2	2
WED18.05	2018-08-13	50.151084	-122.79599	2099	Wedge Mountain	1	1

Supplementary figures

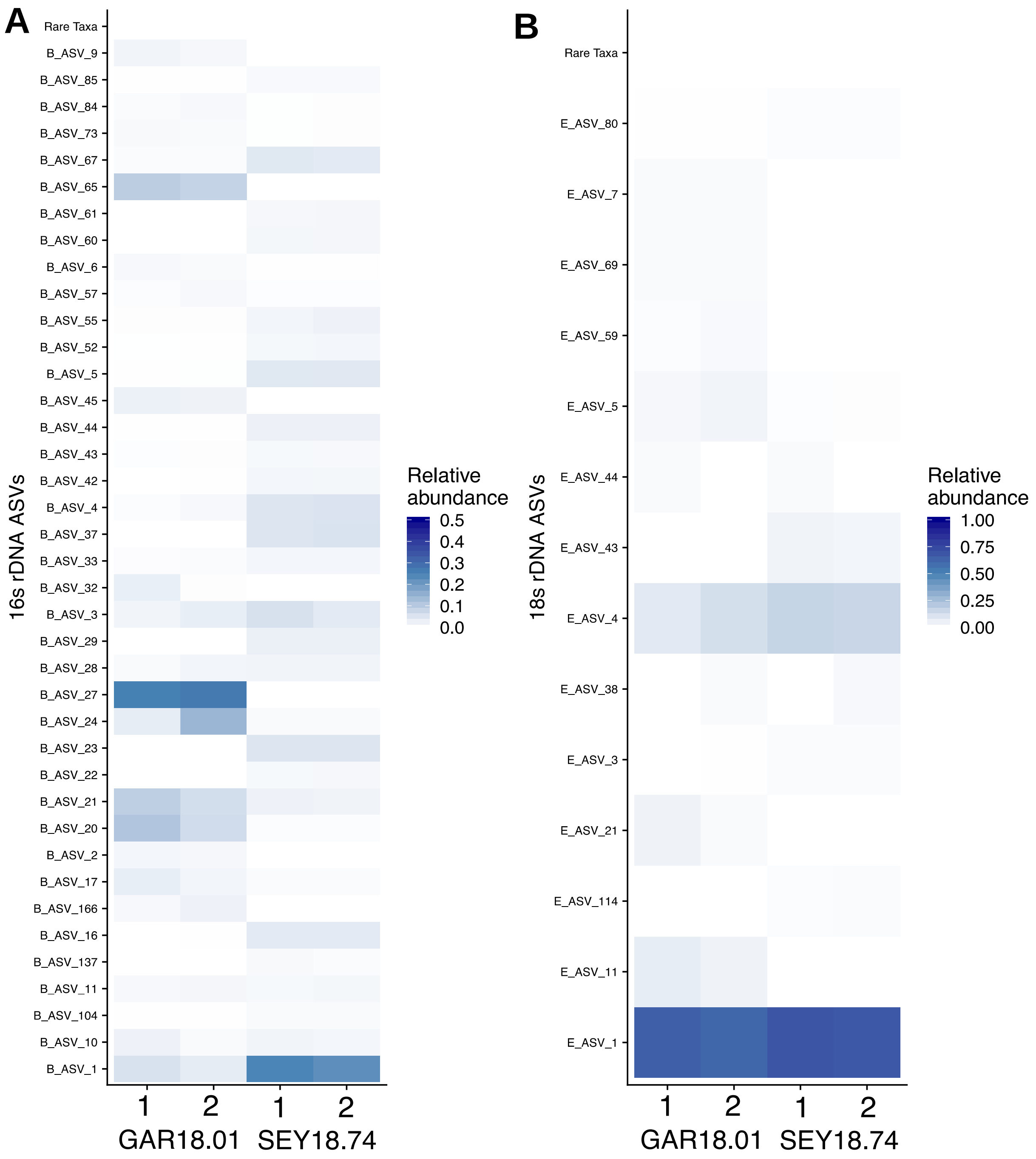

Supplementary Figure 1: Heatmaps of bacteria (A) and eukaryote (B) ASVs that were at least 1% relatively abundant in the samples that were replicated with both cell lysis methods. The numbers on the x-axis indicate the cell lysis method and respective sequencing run the profile is from in each sample.

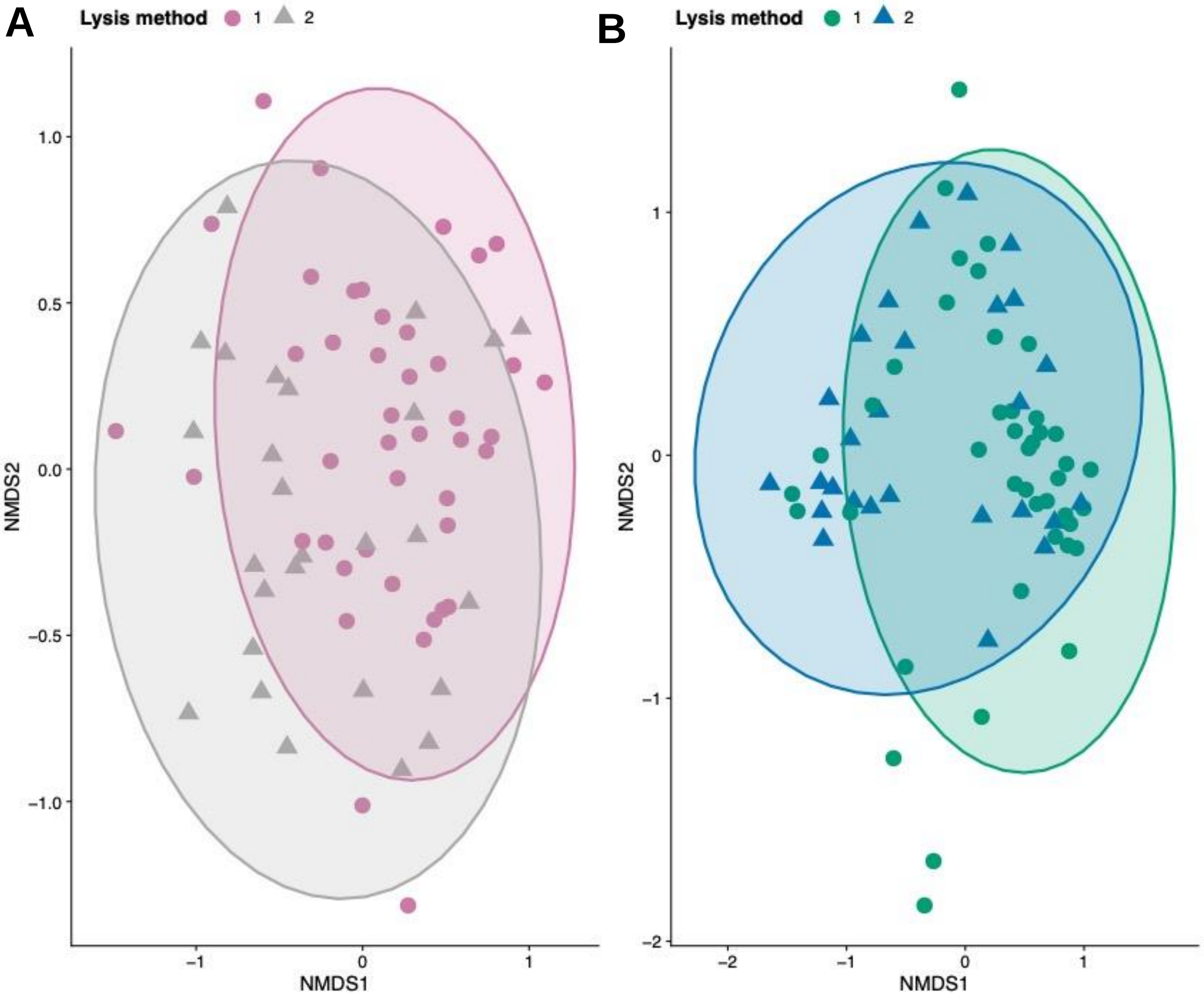

Supplementary Figure 2: NMDS plots of the 16S rDNA relative abundances with samples highlighted to contrast the two different lysis methods used. (A) and 18s rDNA (B) ASV relative abundances where each point represents a sample and they are grouped by which lysis method was done on it. The stress values are 0.2 (A) and 0.18 (B).

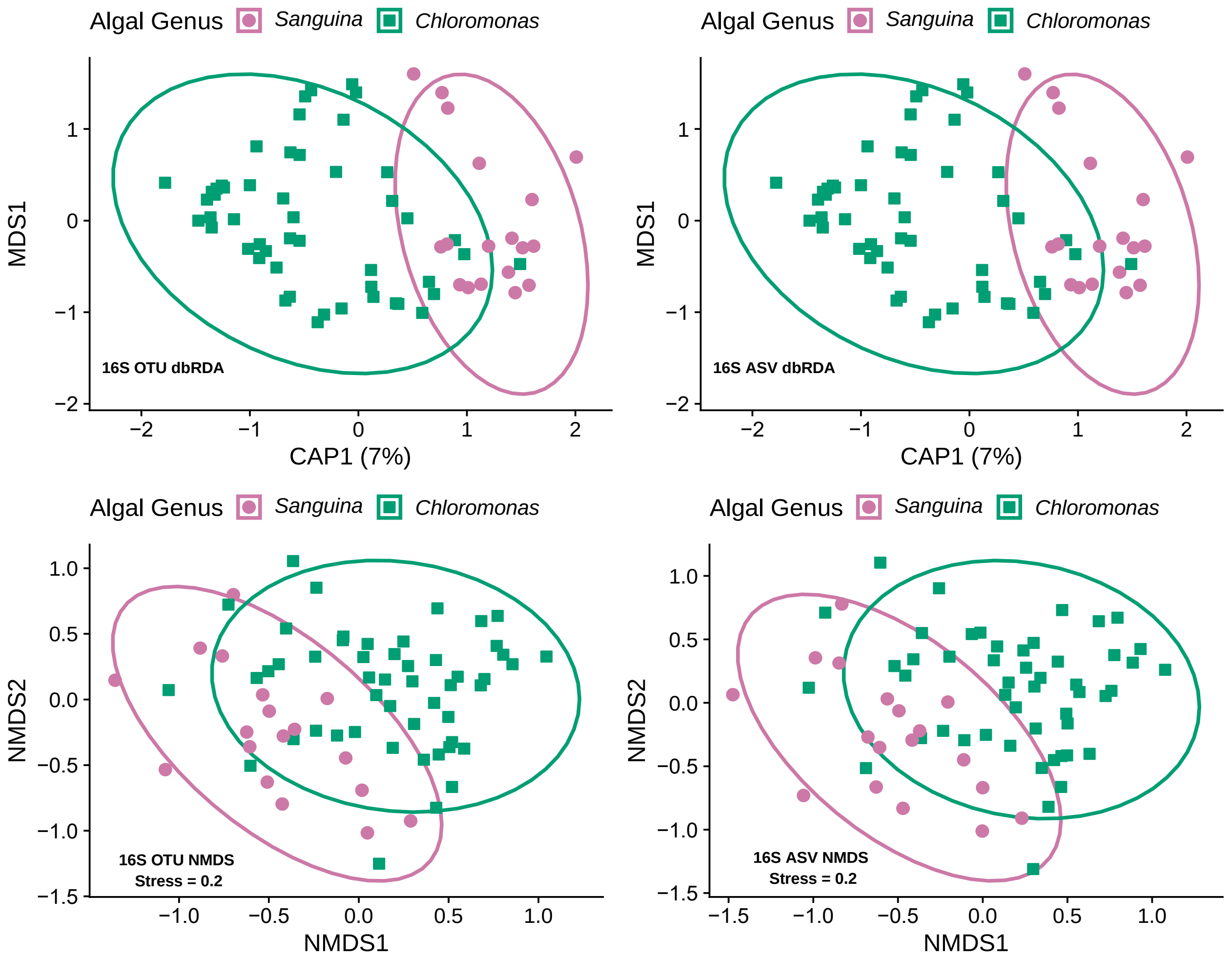

Supplementary Figure 3: NMDS and dbRDA plots of the bacterial 16S relative abundances for ASVs and OTUs respectively. The dbRDAs were constrained by the dominant algal genus found in that sample. Each sample was classified as either Chloromonas- or Sanguina-dominant, based on which genus was found in higher relative abundance.

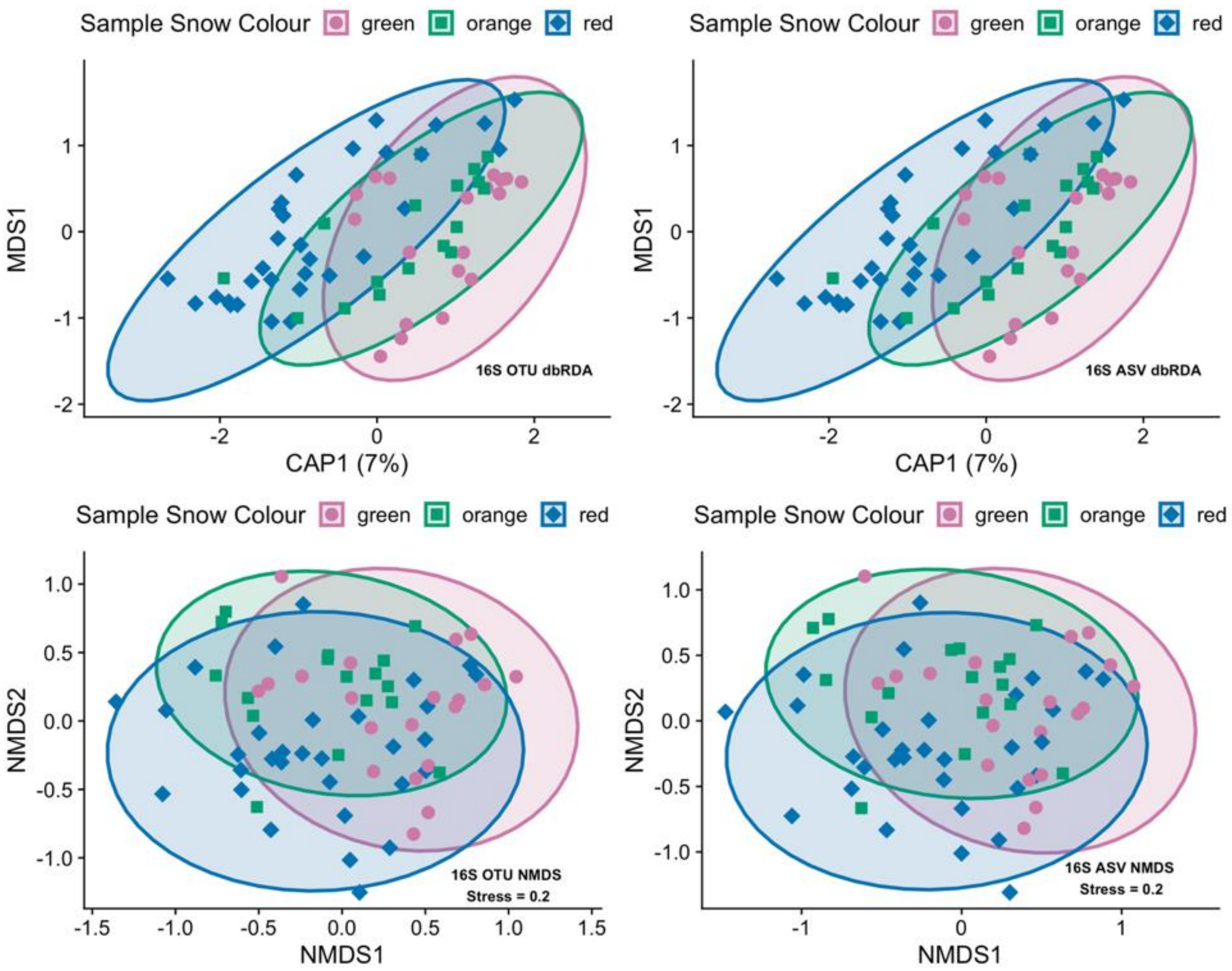

Supplementary Figure 4: NMDS and dbRDA plots of the bacterial 16S relative abundances for ASVs and OTUs respectively. The dbRDAs were constrained by the colour of snow the sample was taken from.

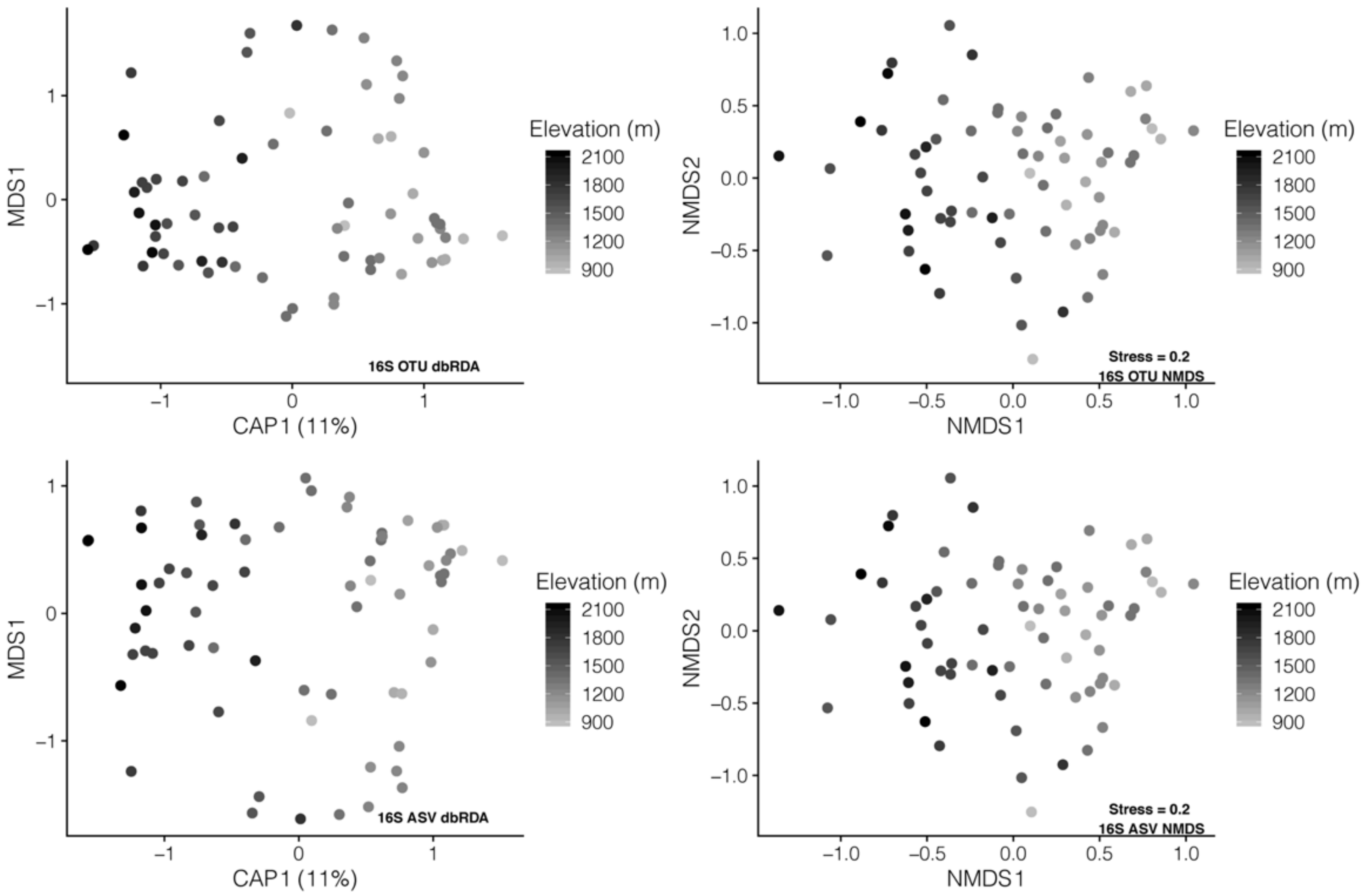

Supplementary Figure 5: NMDS and dbRDA plots of the bacterial 16S relative abundances for ASVs and OTUs respectively. The dbRDAs were constrained by the elevation the sample was taken from.

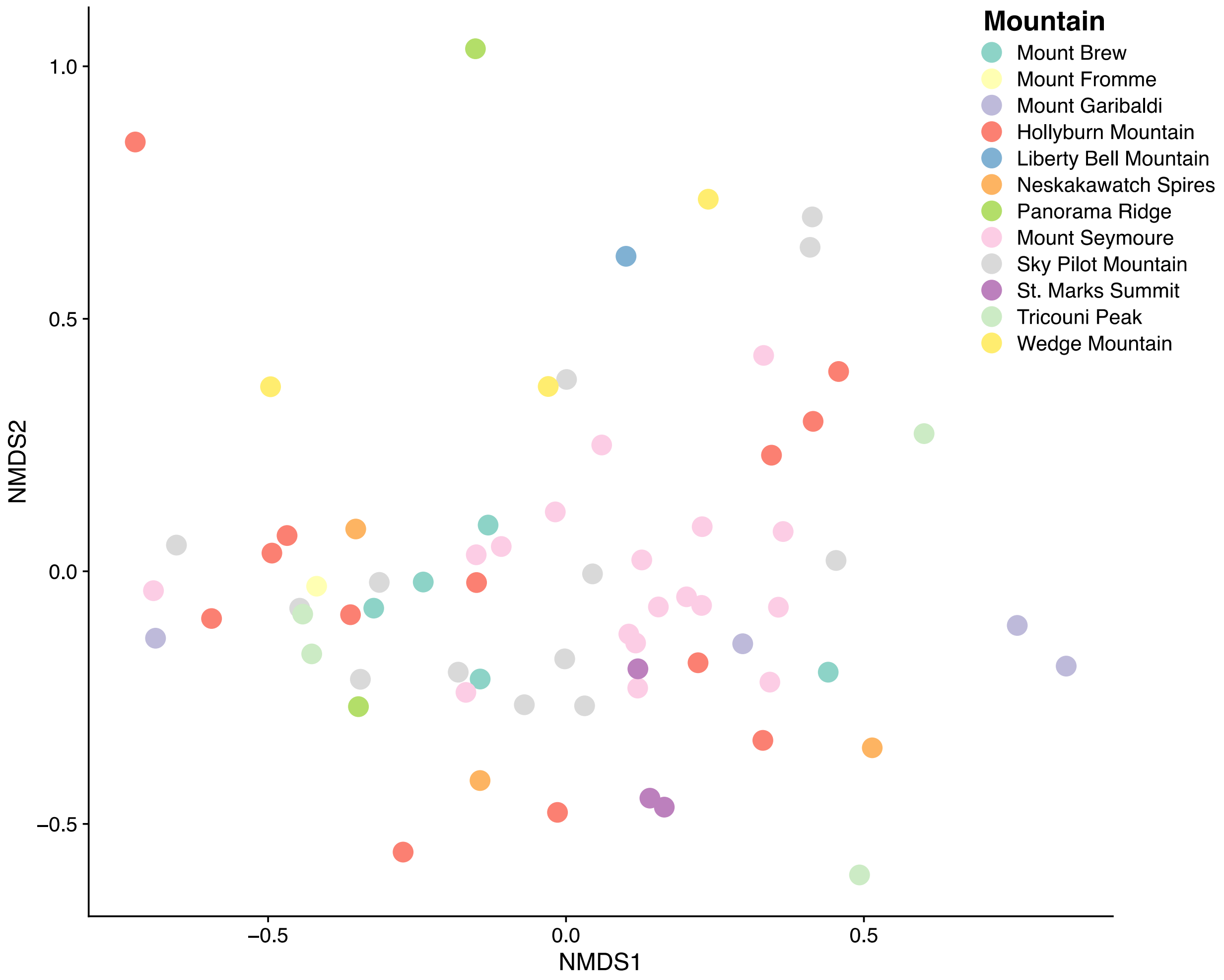

Supplementary Figure 6: NMDS ordination of the 16S metabarcoding data from all 68 samples, and each point is colored by which mountain it was collected on. Stress value is 0.19.

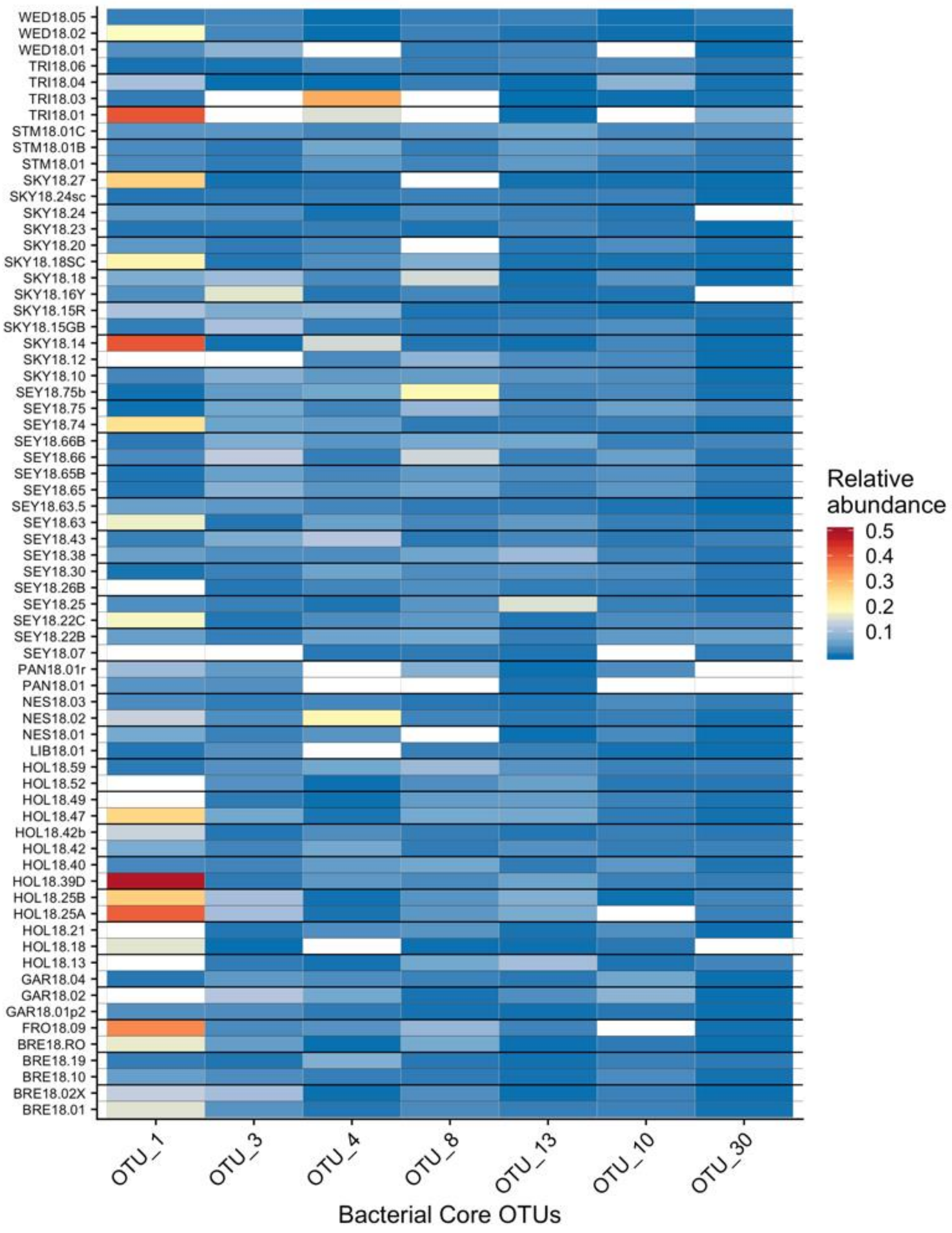

Supplementary Figure 7: A heatmap of the relative abundances of the 7 widespread OTUS detected in each sample. Values of 0, where the OTU was undetected in a sample, are coloured white.

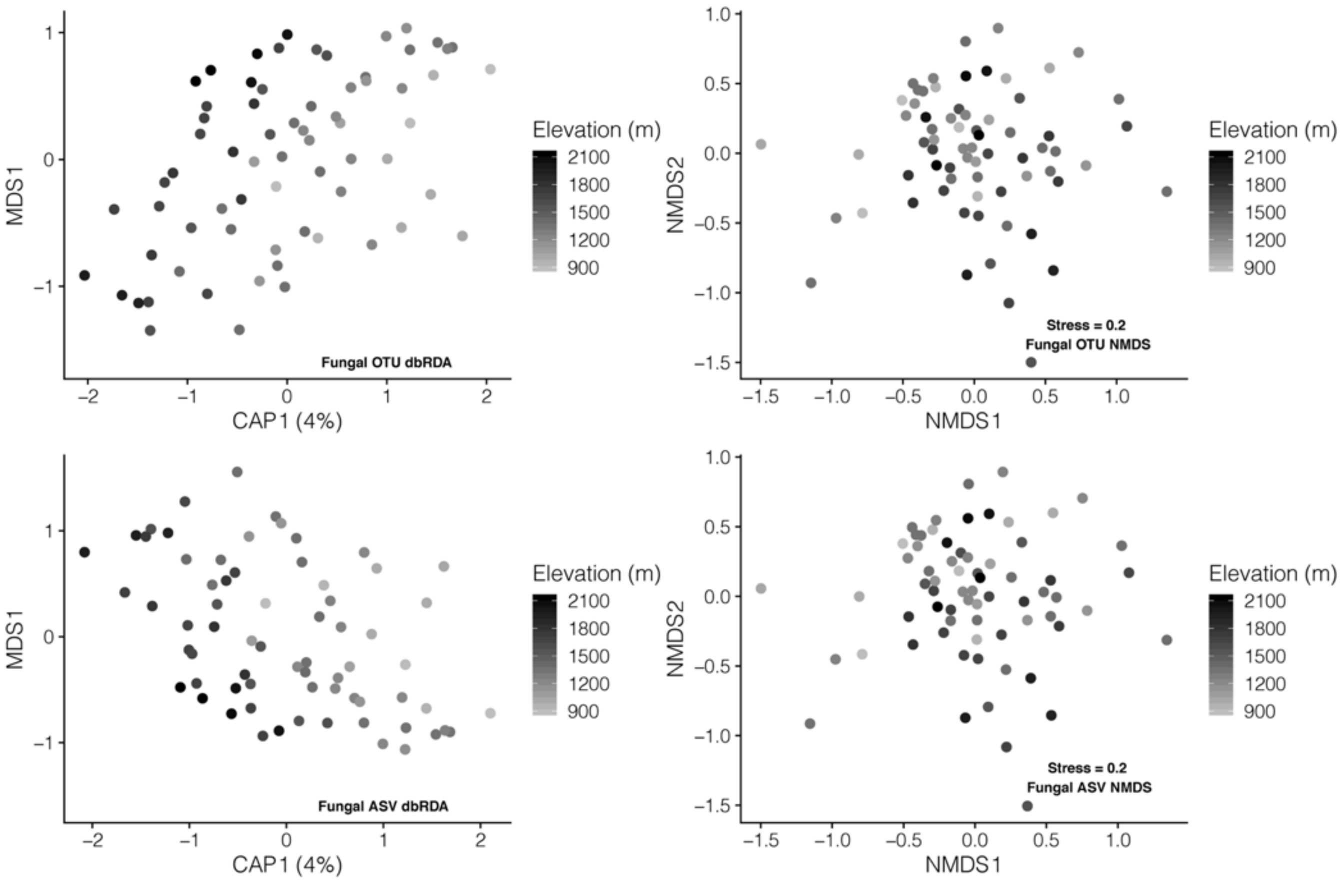

Supplementary Figure 8: NMDS and dbRDA plots of the bacterial fungal 18S relative abundances for ASVs and OTUs respectively. The dbRDAs were constrained by the colour of snow the sample was taken from.

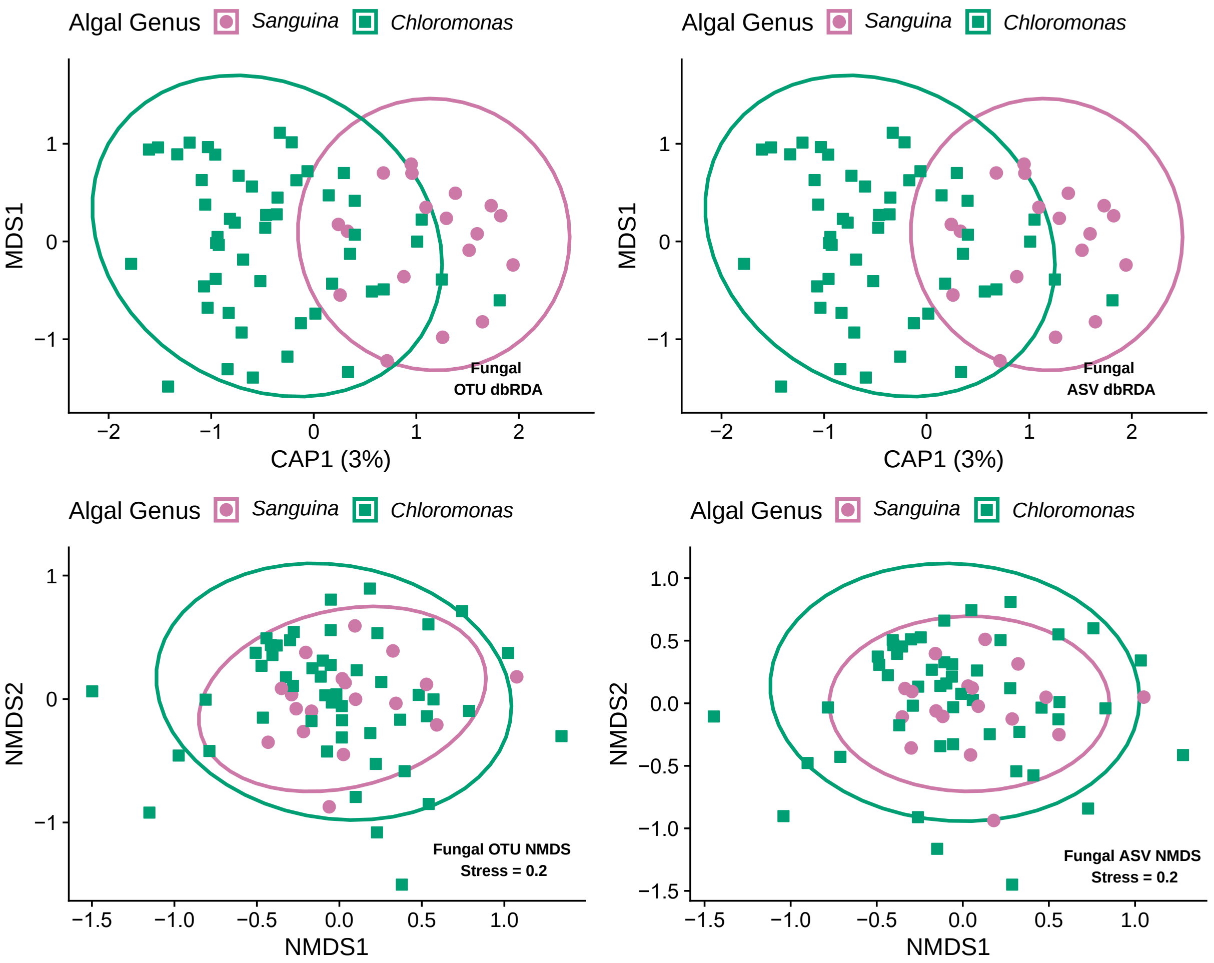

Supplementary Figure 9: NMDS and dbRDA plots of the bacterial fungal 18S relative abundances for ASVs and OTUs respectively. The dbRDAs were constrained by the dominant algal genus found in each sample. Each sample was classified as either Chloromonas- or Sanguina- dominant, based on which genus was found in higher relative abundance.

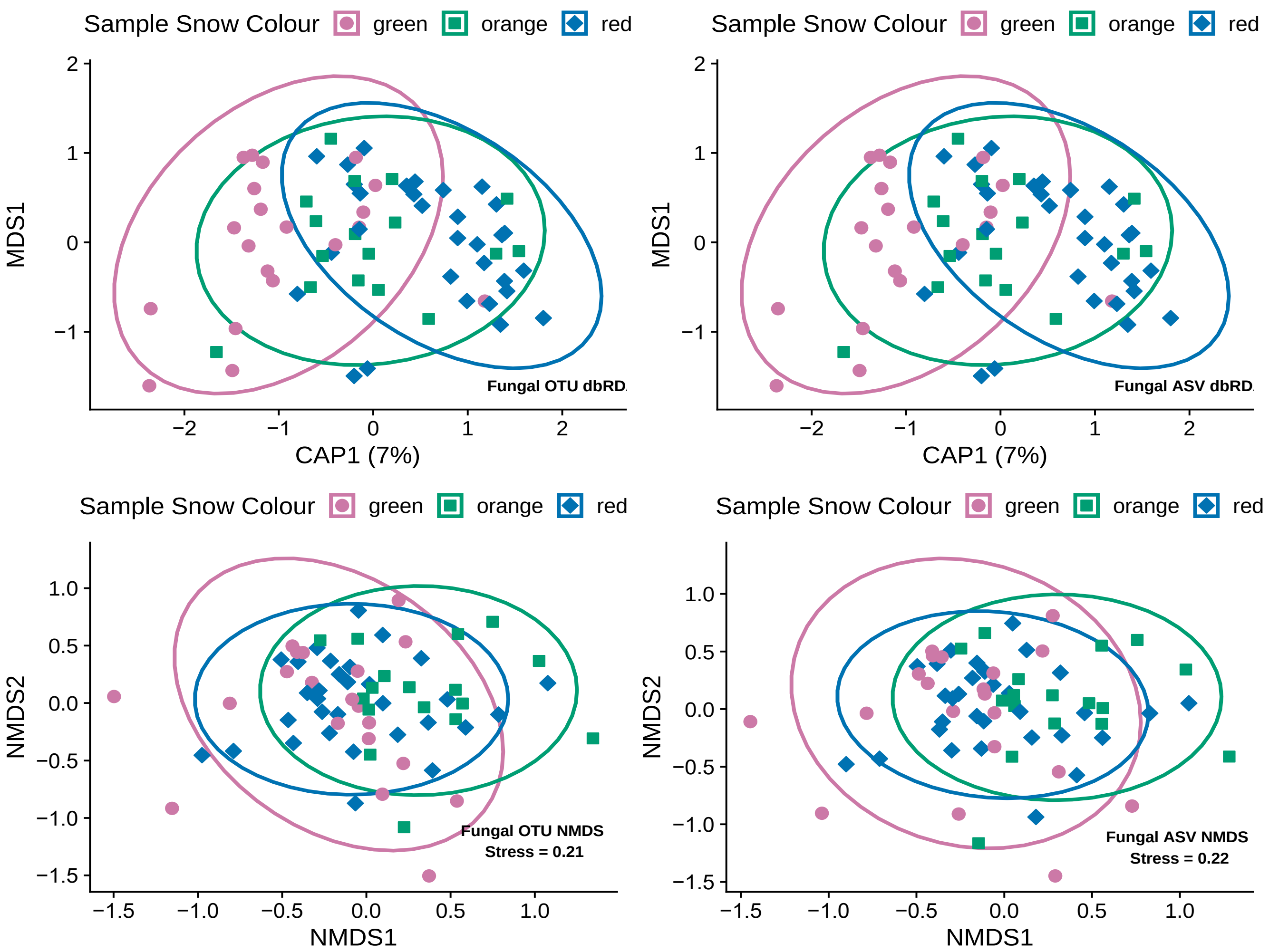

Supplementary Figure 10: NMDS and dbRDA plots of the bacterial fungal 18S relative abundances for ASVs and OTUs respectively. The dbRDAs were constrained by the colour of snow the sample was taken from.

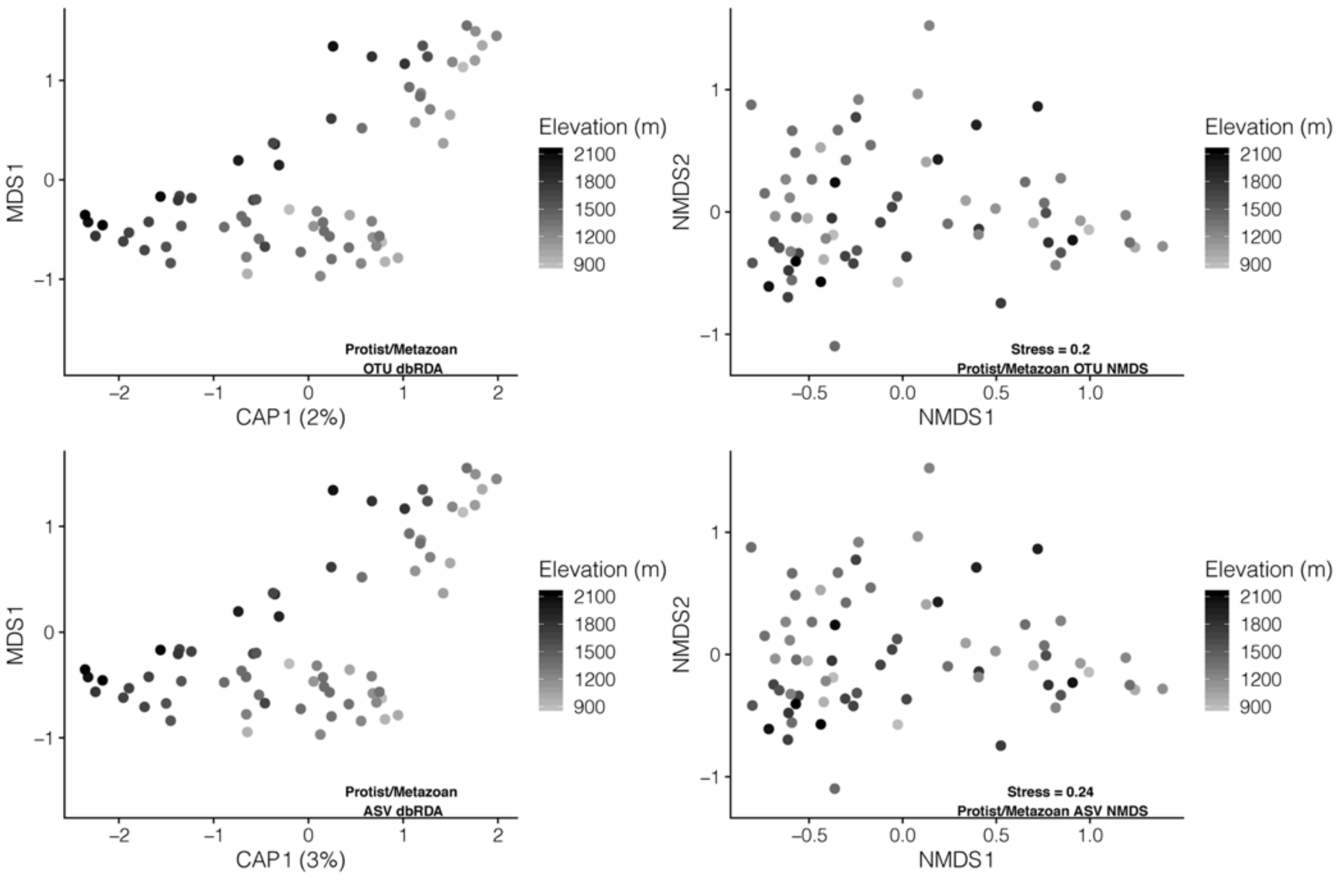

Supplementary Figure 11: NMDS and dbRDA plots of the protist/metazoan 18S relative abundances for ASVs and OTUs respectively. The dbRDAs were constrained by the colour of snow the sample was taken from.

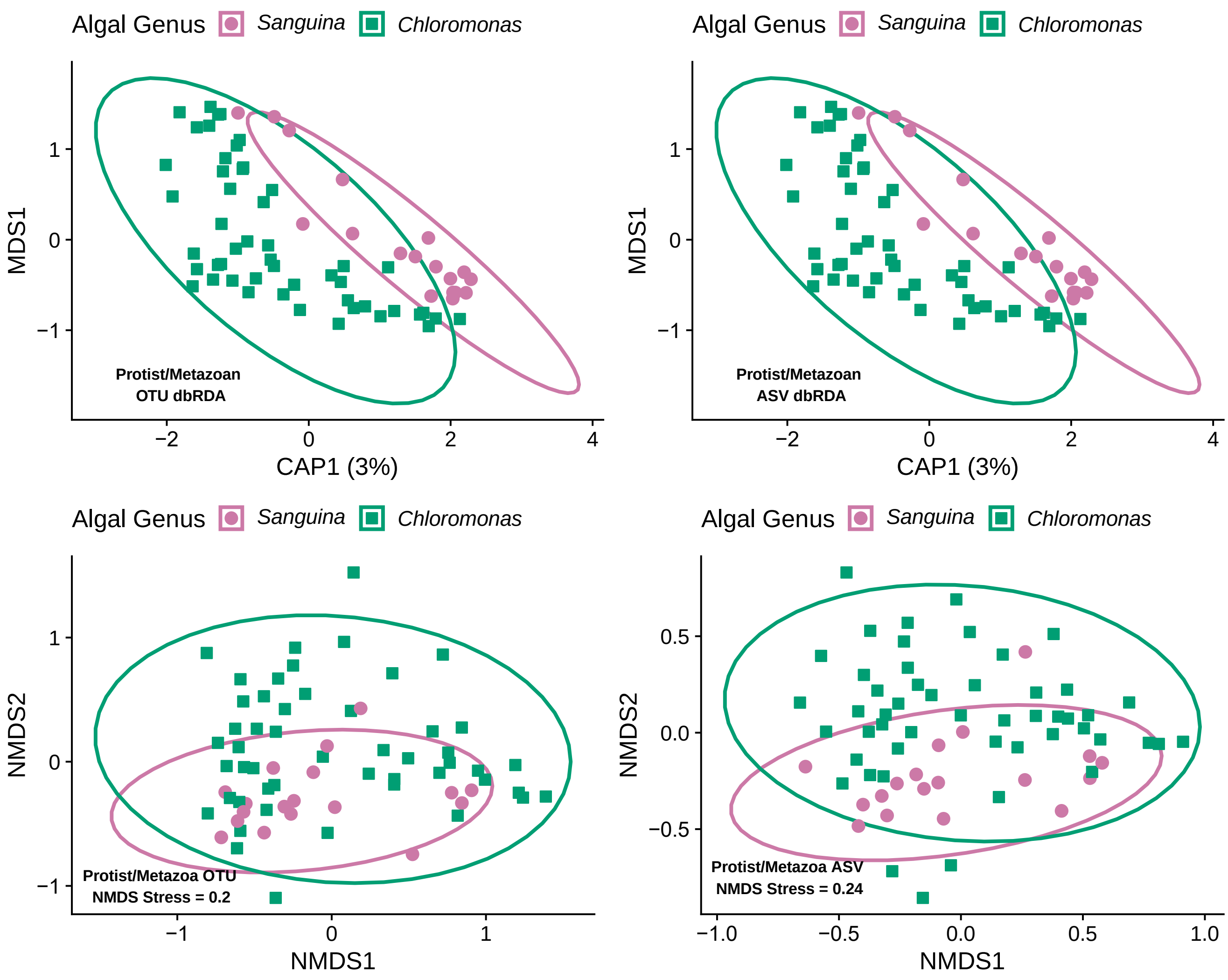

Supplementary Figure 12: NMDS and dbRDA plots of the bacterial protist/metazoan 18S relative abundances for ASVs and OTUs respectively. The dbRDAs were constrained by the dominant algal genus found in each sample.

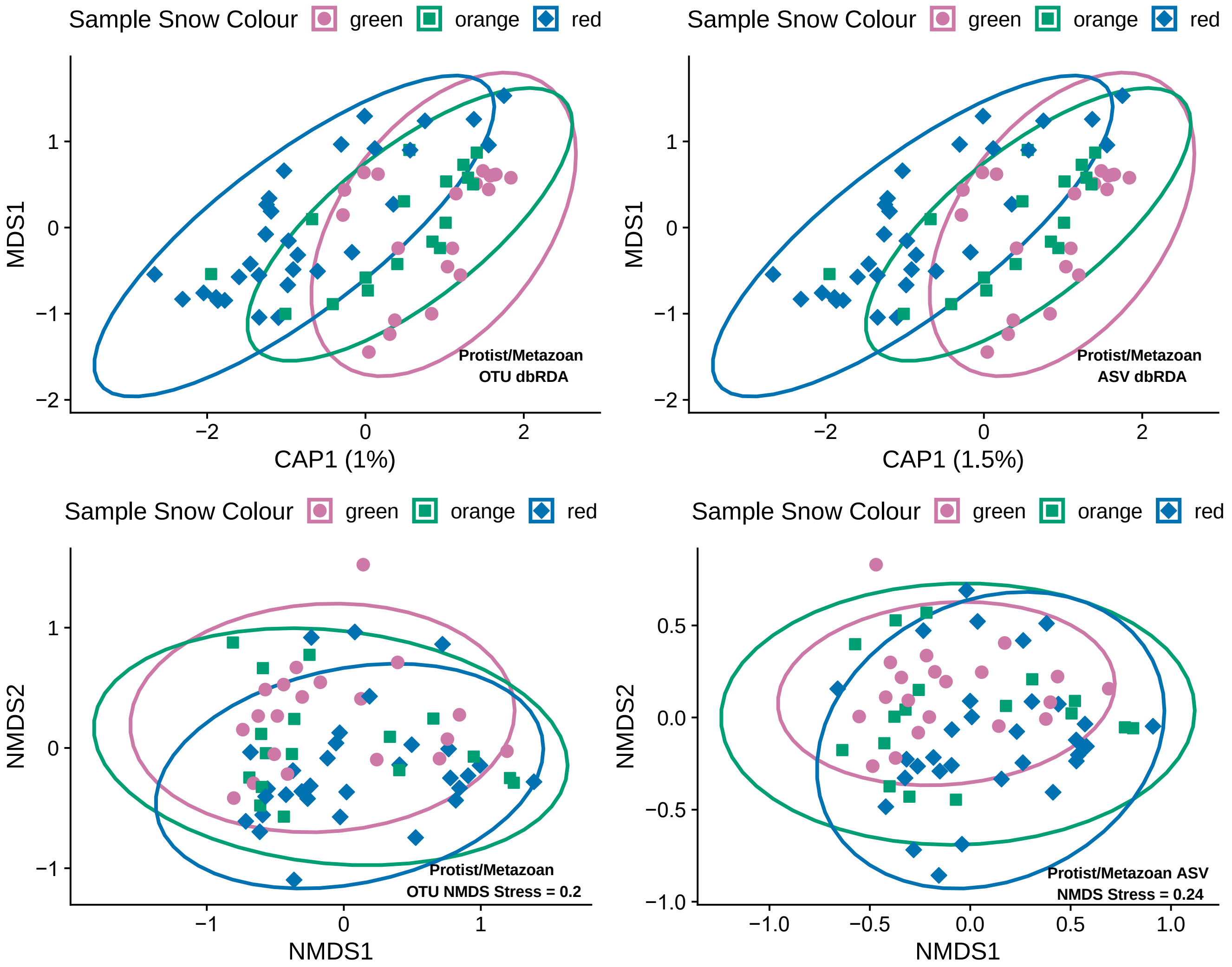

Supplementary Figure 13: NMDS and dbRDA plots of the bacterial protist/metazoan 18S relative abundances for ASVs and OTUs respectively. The dbRDAs were constrained by the colour of snow the sample was taken from.

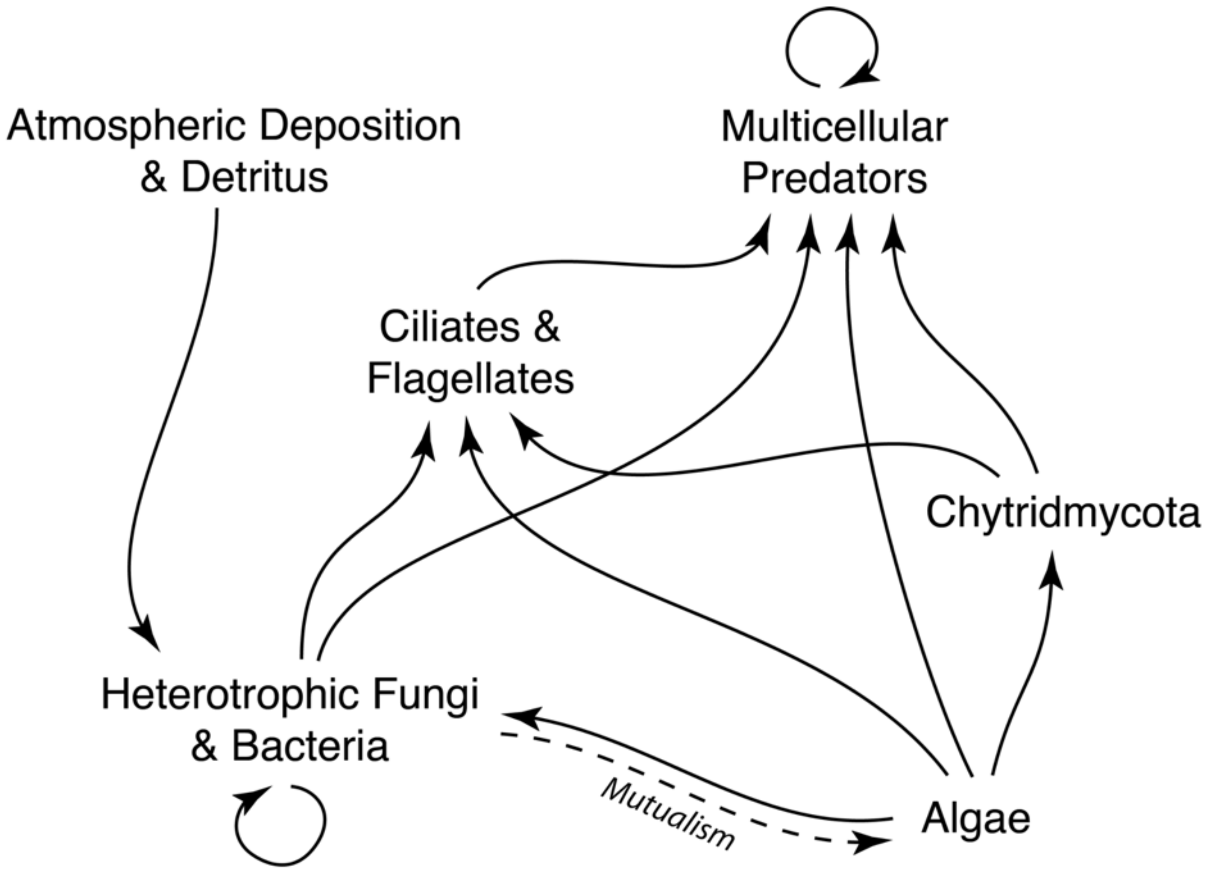

Supplementary Figure 14: Hypothetical food web within the snow algae microbiome based on organisms and interactions seen in photomicrographs. Black arrows represent the flow of C to higher trophic levels, and the dotted lines represent hypothetical mutualistic feedback between trophic levels. Death and lysis of organisms that then feedback into the foodweb through decomposition by detritovores is represented here as "Detritus".
